# Blocking TRPM4 alleviates pancreatic acinar cell damage via an NMDA receptor-dependent pathway in acute pancreatitis

**DOI:** 10.7150/thno.116520

**Published:** 2025-06-09

**Authors:** Yifan Ren, Qing Cui, Wuming Liu, Hangcheng Liu, Tao Wang, Hongwei Lu, Yi Lv, Rongqian Wu

**Affiliations:** 1 National Local Joint Engineering Research Center for Precision Surgery & Regenerative Medicine, Shaanxi Provincial Center for Regenerative Medicine and Surgical Engineering, First Affiliated Hospital of Xi'an Jiaotong University. Xi'an, Shaanxi Province, China.; 2 Department of General Surgery, The Second Affiliated Hospital of Xi'an Jiaotong University, Xi'an, Shaanxi Province, China.; 3 Department of Cardiology, Xi'an Third Hospital Affiliated to Northwest University, Xi'an, Shaanxi Province, China.; 4 Department of Hepatobiliary Surgery, First Affiliated Hospital of Xi'an Jiaotong University, Xi'an, Shaanxi Province, China.

**Keywords:** acute pancreatitis, intracellular Ca^2+^, TRPM4/NMDARs, mitochondrial dysfunction, ER stress

## Abstract

**Background:** Mitochondrial dysfunction caused by Ca^2+^ overload in pancreatic acinar cells is an important mechanism in the pathogenesis of acute pancreatitis (AP). Transient receptor potential cation channel melastatin 4 (TRPM4), a non-selective cation channel, can be activated by intracellular Ca^2+^, and is involved in mediating damage to neuronal mitochondrial function. However, the role of TRPM4 activation in mitochondrial dysfunction during AP remains unknown.

**Methods:** We employed three mouse models of AP (intraperitoneal administration of L-arginine, cerulein plus lipopolysaccharides (LPS), or cerulein alone) for *in vivo* studies. For *in vitro* studies, cerulein+ LPS was used to induce mitochondrial dysfunction and cell death in AR42J cell. *Trpm4* gene-defective mice and plasmids were utilized to downregulate the expression of TRPM4 in mice or overexpress TRPM4 in AR42J. 9-Phenanthrol, a specific inhibitor of TRPM4, was used to antagonize TRPM4 activity both *in vitro* and *in vivo*.

**Results:** Pancreatic TRPM4 levels were increased in all three AP models. Blocking TRPM4 activity with 9-phenanthrol or knocking down TRPM4 expression alleviated pancreatic damage and reduced mortality in AP mice. The protective effect of TRPM4 defects on AP was associated with improved mitochondrial function in pancreatic acinar cells. Mechanistically, TRPM4 activation induced mitochondrial dysfunction and cell death in AP were dependent on the presence of N-methyl-D-aspartate receptors (NMDARs). Blocking NMDARs mitigates the aggravated mitochondrial damage, ER stress and cell death caused by TRPM4 activation in AP.

**Conclusions:** TRPM4 activation contributes to pancreatic acinar cells damage via an NMDAs-dependent pathway in AP. The TRPM4/NMDARs complex provides a new target for the future treatment of AP.

## Introduction

Acute pancreatitis (AP) is a disease characterized by the self-digestion of pancreatic tissue, accompanied by varying degrees of tissue destruction, edema or hemorrhage 1. Alcohol, hyperlipidemia, and cholelithiasis account for approximately 72% of AP cases 2. Severe AP is a non-self-limiting syndrome characterized by pancreatic necrosis and a high risk of multiple organ dysfunction syndrome (MODS), placing a heavy burden on intensive care units 3. Due to the limited understanding of its pathogenesis, palliative treatment is the main treatment for severe AP, and specific treatments are still elusive 4.

Healthy mitochondria play a core role in maintaining the normal homeostasis of pancreatic exocrine acinar cells 5, 6. Many studies have shown that abnormal mitochondrial function is a key factor in AP pathogenesis 7-9. Mitochondrial damage caused by a variety of pathogenic factors can lead to impaired autophagy and subsequently cause lipid metabolism disorders and endoplasmic reticulum stress (ER stress) in AP 10. Our previous studies have shown that impaired mitochondrial dynamics induces oxidative stress in pancreatic cell, aggravate the unfolded protein response (UPR), promote cell apoptosis and trigger an inflammatory response 11-13. Therefore, restoring impaired mitochondrial function in AP provides a rationale for developing targeted therapeutic options.

Calcium (Ca^2+^) overload is a key contributor to exocrine acinar cells injury in AP 14. Transient receptor potential cation channel melastatin 4 (TRPM4) is a non-selective cation channel that can be activated by increasing the concentration of cytoplasmic Ca^2+^ 15-17. TRPM4 activation causes mitochondrial injury and cell death in the nervous system when TRPM4 is physically coupled with N-methyl-d-aspartate receptors (NMDARs), a class of glutamate-gated, calcium-permeable neurotransmitter receptors 18. Both NMDARs and TRPM4 are expressed in the pancreas. However, the role of TRPM4 activation and its interaction with NMDARs in pancreatic cell injury during AP remains unknown. Therefore, we hypothesized that TRPM4 activation leads to pancreatic cell damage via an NMDAs-dependent pathway in AP. The aim of this study was to determine whether pancreatic cell injury in AP is related to TRPM4 activation and, if so, the role of TRPM4 and NMDARs interactions in this process. We investigated whether TRPM4 is involved in the association between elevated intracellular Ca^2+^ levels and mitochondrial dysfunction. This study provides mechanistic insight for the development of targeted treatments for AP.

## Results

### TRPM4 expression is increased in various AP models

**Figure 1:**
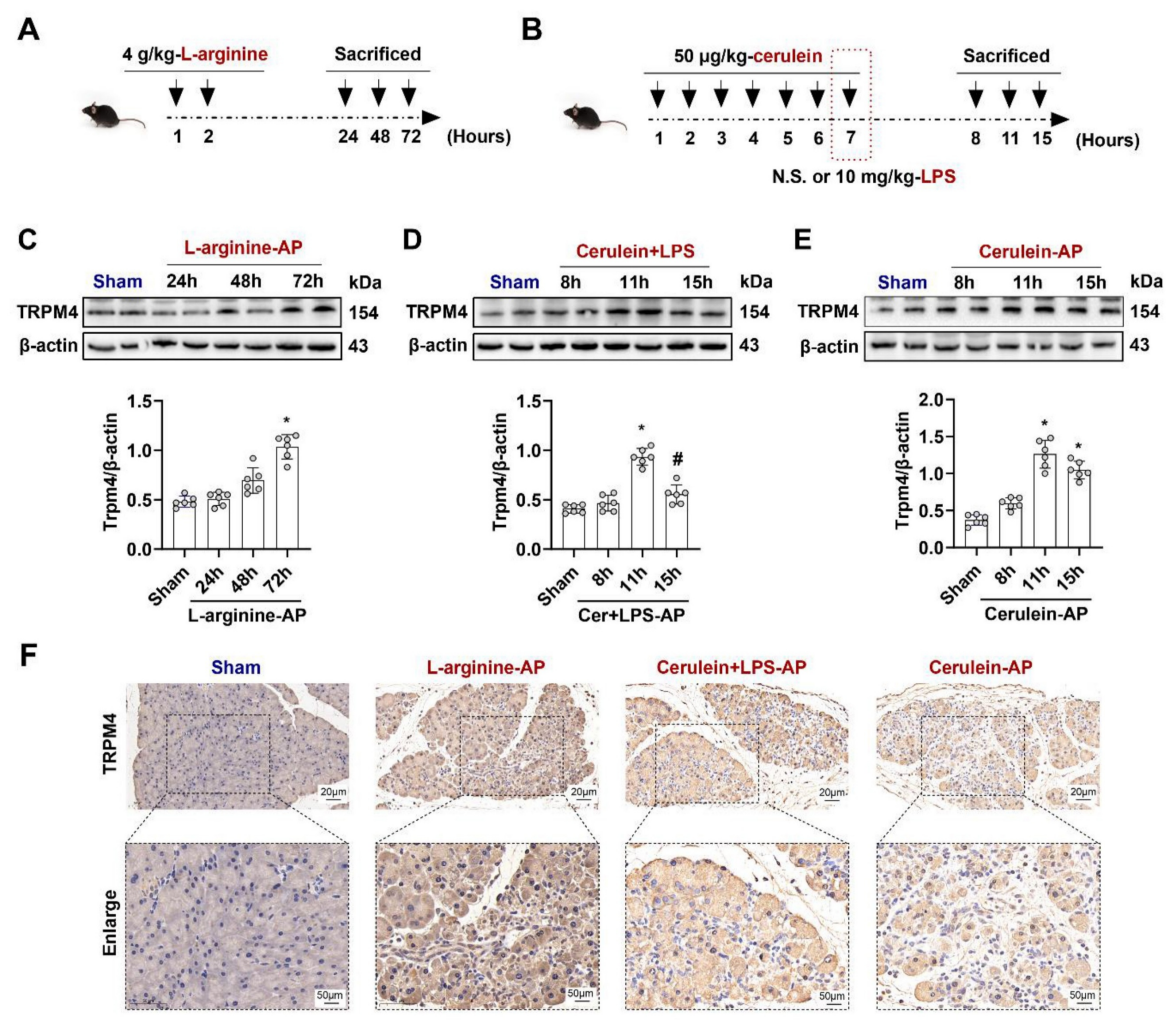
** TRPM4 expression levels increased in various AP models.** (**A**) Schematic diagram of L-arginine-AP animal model; (**B**) Schematic diagram of Cerulein+ LPS- or Cerulein-AP animal model; (**C-E**) Western blot analysis the TRPM4 expression level in the pancreas; (**F**) Representative photos of TRPM4 staining of the pancreas (200X or 400X). n = 6, Error bars indicate the SEM; ∗ P < 0.05 vs Sham; # P < 0.05 vs 11h in cerulein + LPS - AP. TRPM4, Transient receptor potential cation channel melastatin 4; AP, acute pancreatitis; LPS, lipopolysaccharide; N.S., normal saline.

To elucidate the role of TRPM4 in AP, we first examined changes in TRPM4 expression levels in the pancreas. As shown in Figures 1A-B, L-arginine or Cerulein+ lipopolysaccharides (LPS) or Cerulein alone were used to induce different mouse AP models, respectively (Detailed description is given in the *Materials and Methods*). The expression level of TRPM4 in the mouse pancreas was almost unchanged within 48 h after the intraperitoneal injection of L-arginine; however, it increased to approximately 1.5 times than the base level at 72 h (P < 0.05, Figure 1C). A signally increase in TRPM4 expression was observed in mice at 11 h after AP induction with cerulein+ LPS, and the TRPM4 level returned to the baseline level at 15 h (P < 0.05, Figure 1D). Furthermore, the expression trend of TRPM4 in cerulein- treated AP model mice was similar to that in cerulein+ LPS-induced AP model mice (Figure 1E), suggesting that the two models may share the same pathogenesis. Figure 1F shows the TRPM4 expression in the pancreatic tissue of AP model mice and control mice. The destruction of pancreatic tissue combined with the deepening of TRPM4 staining confirmed the conclusions obtained in Figures 1C-E.

### 9-phenanthrol protects against pancreatic injury in experimental AP

**Figure 2:**
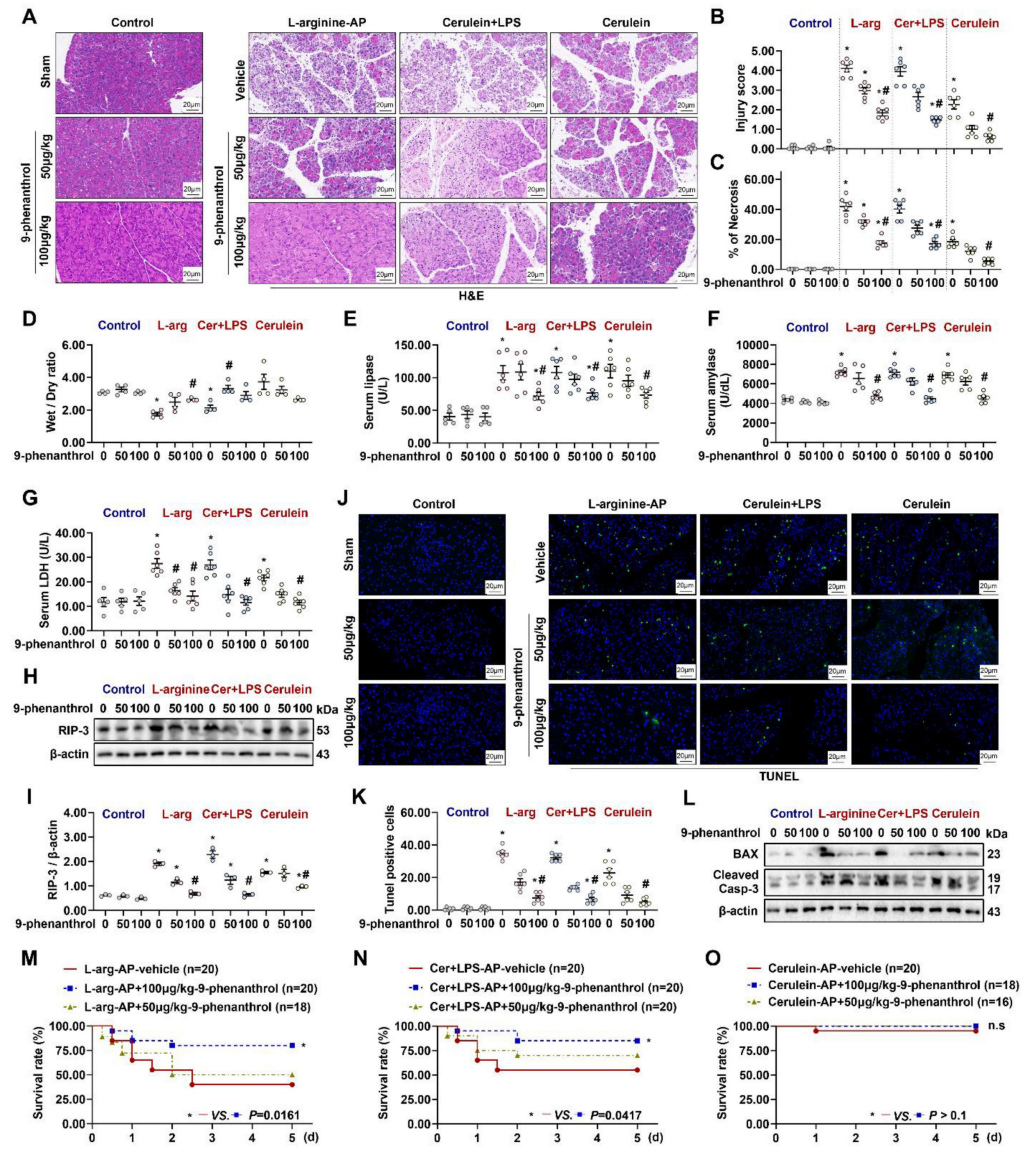
** 9-phenanthrol administration was protective in experimental AP.** (**A**) Representative images of H&E staining of the pancreas (200X); (**B**) Pancreatic injury scores; (**C**) Percentages of necrotic areas; (**D**) Pancreatic Wet/Dry ratio; (**E**) Serum lipase levels; (**F**) Serum amylase levels; (**G**) Serum LDH levels; (**H-I**) Western blot analysis and quantitative of the RIP3 expression level in the pancreas; (**J**) Representative images of TUNEL staining (200X); (**K**) Quantitative of TUNEL staining; (**L**) Western blot analysis of the BAX and Cleaved Caspase-3 expression level in the pancreas; (**M**) 5-day survival of L-arginine-AP mice; (**N**) 5-day survival of Cerulein + LPS-AP mice; (**O**) 5-day survival of Cerulein-AP mice. n = 3-20, Error bars indicate the SEM; ∗ P < 0.05 vs Sham; # P < 0.05 vs Vehicle. RIP3, receptor-interacting protein kinase 3; LDH, lactate dehydrogenase; TUNEL, TdT-mediated dUTP Nick-End Labeling; AP, acute pancreatitis; LPS, lipopolysaccharide.

Pathological examination revealed the role of TRPM4 in AP development. As shown in Figures 2A-C & Figures S1A-C, L-arginine-, cerulein+ LPS-, cerulein-induced AP caused different degrees of pancreatic damage in mice. Among them, cerulein-induced AP was less severe than L-arginine- or cerulein+ LPS-induced AP. The intraperitoneal injection of the TRPM4 inhibitor 9-phenanthrol alleviated or eliminated pancreatic injury and necrosis in the three mouse models of AP. 9-phenanthrol exhibited a dose-dependent protective effect on the pancreas (P < 0.05, Figures 2A-C; P > 0.05, Figures S1A - C).

Severe AP is often accompanied by pancreatic hemorrhage, necrosis, and macroscopic reductions in pancreatic volume and weight. Mild AP, also known as edematous pancreatitis, is associated with or without weight gain due to congestive edema of the pancreas 1. As shown in Figure 2D, L-arginine-AP and cerulein+ LPS-AP caused a decrease in pancreatic water content, suggesting pathological atrophy of the pancreas (P < 0.05). 9-phenanthrol gradually normalized the abnormal wet weight /dry weight ratio of the pancreas (Figure 2D). Cerulein-AP model mice showed increased pancreatic edema, and 100μg/kg of 9-phenanthrol alleviated cerulein-induced pancreatic edema, but there are no sings of significance on Figure 2D. Similarly, level of serum LDH, amylase, and lipase, which are associated with pancreatic acinar cells injury, were also elevated in all three AP models. The application of 9-phenanthrol reduced abnormally elevated serum LDH, amylase and lipase levels (P < 0.05, Figures 2E-G).

The expression of RIP-3, a necrosis-related protein, upregulated in pancreatic tissues of multiple AP models and downregulated after 9-phenanthrol administration (Figures 2H-I). The protective effect of TRPM4 inhibition against apoptosis in pancreatic tissues of AP mice was confirmed by TUNEL staining (P < 0.05, Figures 2J-K). Similarly, the expression level of apoptosis-related proteins, i.e., BAX and cleaved caspase-3, which was highly expressed in this AP models, was downregulated after the intraperitoneal injection of 100 μg/kg 9-phenanthrol (Figure 2L).

To further evaluate the protective ability of 9-phenanthrol against AP, a 5-day survival experiment was performed. As shown in Figure 2M, among the 20 L-arginine-induced AP model mice, 7 died on the first day (3 died within 12 h after the procedure). Five died successively over the next 1-2 days. At the end of the experiment (day 5), 12 of the 20 mice had died, resulting in a survival rate of 40%. After treatment with 50 μg/kg 9-phenanthrol, 9 of 18 L-arginine-induced AP model mice died within 2 days; the survival rate of the mice was 50% at the end of the experiment (P > 0.05). However, 100 μg/kg 9-phenanthrol increased the survival rate of L-arginine-AP model mice to almost 78% (P < 0.05). Similarly, 100 μg/kg 9-phenanthrol increased the survival rate of cerulein+ LPS-induced AP model mice from approximately 50% to more than 80% (P < 0.05, Figure 2N). Cerulein-induced AP only caused the death of one experimental animal (Within day 1), and no death was observed after the application of 9-phenanthrol (P > 0.05, Figure 2O). These results suggest that cerulein-AP is a mild and self-limiting disease and that the damage only manifests at the organ level, with little systemic effect.

Inflammation is another pathological feature of AP 7. In this study, neutrophils were labeled with LY6G to visually observe the changes in the number of inflammatory cells in pancreas. As shown in Figures S2A-B, the intraperitoneal injection of 9-phenanthrol reduced the number of neutrophils in pancreas in AP animal models. Similarly, the elevated levels of IL-6 and TNF-α in the serum of AP mice were also reduced after the application of 9-phenanthrol (Figures S2C-D, P < 0.05).

### 9-Phenanthrol alleviates mitochondrial dysfunction in experimental AP

**Figure 3:**
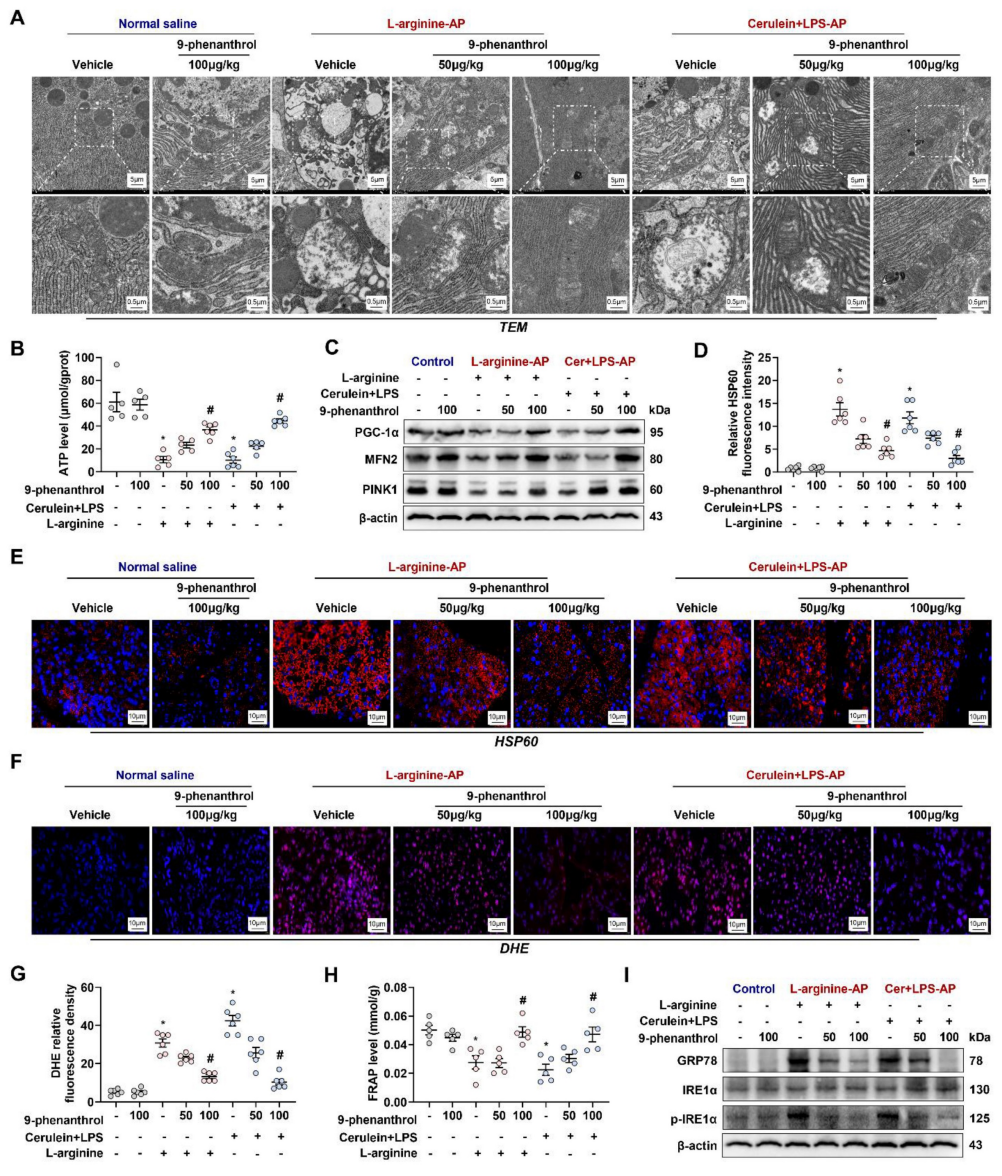
** 9-phenanthrol improved mitochondrial function, decreased oxidative stress and alleviates ER stress in experimental AP.** (**A**) Ultrastructural alterations in the pancreas; (**B**) ATP levels in the pancreas; (**C**) Western blot analysis of the PGC-1α, Mfn-2 and PINK1 expression level in the pancreas; (**D-E**) Representative images and relative fluorescence intensity of HSP60 fluorescence staining in the pancreas (600X); (**F-G**) Representative images and relative fluorescence intensity of DHE staining in the pancreas (600X); (**H**) FRAP levels in the pancreatic tissue; (**I**) Western blot analysis of the GRP78, phosphor-IRE1α and IRE1α expression level in the pancreas. n = 6, Error bars indicate the SEM; ∗ P < 0.05 vs Sham group or vs control; # P < 0.05 vs Vehicle group. PGC-1α, peroxisome proliferative activated receptor-γ coactivator 1α; PINK1, PTEN induced putative kinase 1; DHE, Dihydroethidium; LPS, lipopolysaccharide; FRAP, Ferric Reducing Antioxidant Power.

Mitochondrial damage plays a central role in the pathogenesis of AP 10, 12. When AP occurs, mitochondria in pancreatic cells often exhibit swelling, the disappearance of mitochondrial cristae, and even mitochondrial rupture. AP is accompanied by abnormal mitochondrial function protein expression, oxygen free radical metabolism disorder, and endoplasmic reticulum (ER) stress 10, 13. As shown in Figure 3A, under transmission electron microscopy (TEM), mouse pancreatic cells treated with L-arginine or cerulein+ LPS had abnormal mitochondrial morphology, around which autophagosomes had accumulated. 9-phenanthrol (50 μg/kg) normalized the above mitochondrial morphological abnormalities to a certain extent, while 100 μg/kg 9-phenanthrol basically restored the normal morphology of mitochondria.

As the “energy factory” of the cell, one of the main roles of mitochondria is to produce ATP. Therefore, changes in ATP content directly reflect mitochondrial function. The ATP levels of L-arginine-AP and cerulein+ LPS-AP model mice were reduced to approximately 16% to 17% compared with those of the control mice (Figure 3B). The level was partially restored by treatment with 50 μg/kg 9-phenanthrol (p > 0.05) and restored to 80% to 94% of those in control mice by treatment with 100 μg/kg 9-phenanthrol (p < 0.05). Normal mitochondrial generation, fusion and mitophagy constitute healthy mitochondrial dynamics. PGC-1α is crucial regulators of mitochondrial biogenesis 19. As shown in Figure 3C, western blot analysis showed that PGC-1α expression level was distinctly downregulated in L-arginine- or cerulein+ LPS- AP model mice. Similarly, Mfn-2 and PINK1, regulators of mitochondrial fusion and mitophagy 20, were also decreased in vehicle-treated AP mice. Administration of 9-phenanthrol restored PGC-1α, Mfn-2 and PINK1 levels in AP mice. Thus, 9-phenanthrol treatment restored mitochondrial biogenesis and mitophagy in AP.

HSP60 is a mitochondrial protein whose main function is to assist in the proper transport, folding, and assembly of cellular peptides or proteins 21. HSP60 can be induced by stress, inflammation and immune responses 22, 23. Immunofluorescence showed that HSP60 expression was increased in AP mice, an effect that was reversed by the administration of 9-phenanthrol (Figures 3D-E). Mitochondrial dysfunction leads to an increase in oxygen free radical production and a protective unfolded protein response (UPR) 11. DHE fluorescence staining of reactive oxygen species (ROS) showed that the pancreatic tissue of vehicle treated-AP model mice produced many oxidative stress products (Figures 3F-G), accompanied by a signally decrease in the total antioxidant capacity (P < 0.05, Figure 3H). Western blot analysis showed that the ER stress-related proteins, such as phosphor-IRE1α and GRP78, was also increased in vehicle treated-AP model mice (Figure 3I), suggesting the occurrence of the UPR. The intraperitoneal injection of 9-phenanthrol reduced ROS production, restored the total antioxidant capacity and alleviated ER stress in pancreas of various AP models (P < 0.05, Figures 3F-I).

### Pancreatic damage was alleviated in experimental AP after trpm4 knockout

**Figure 4:**
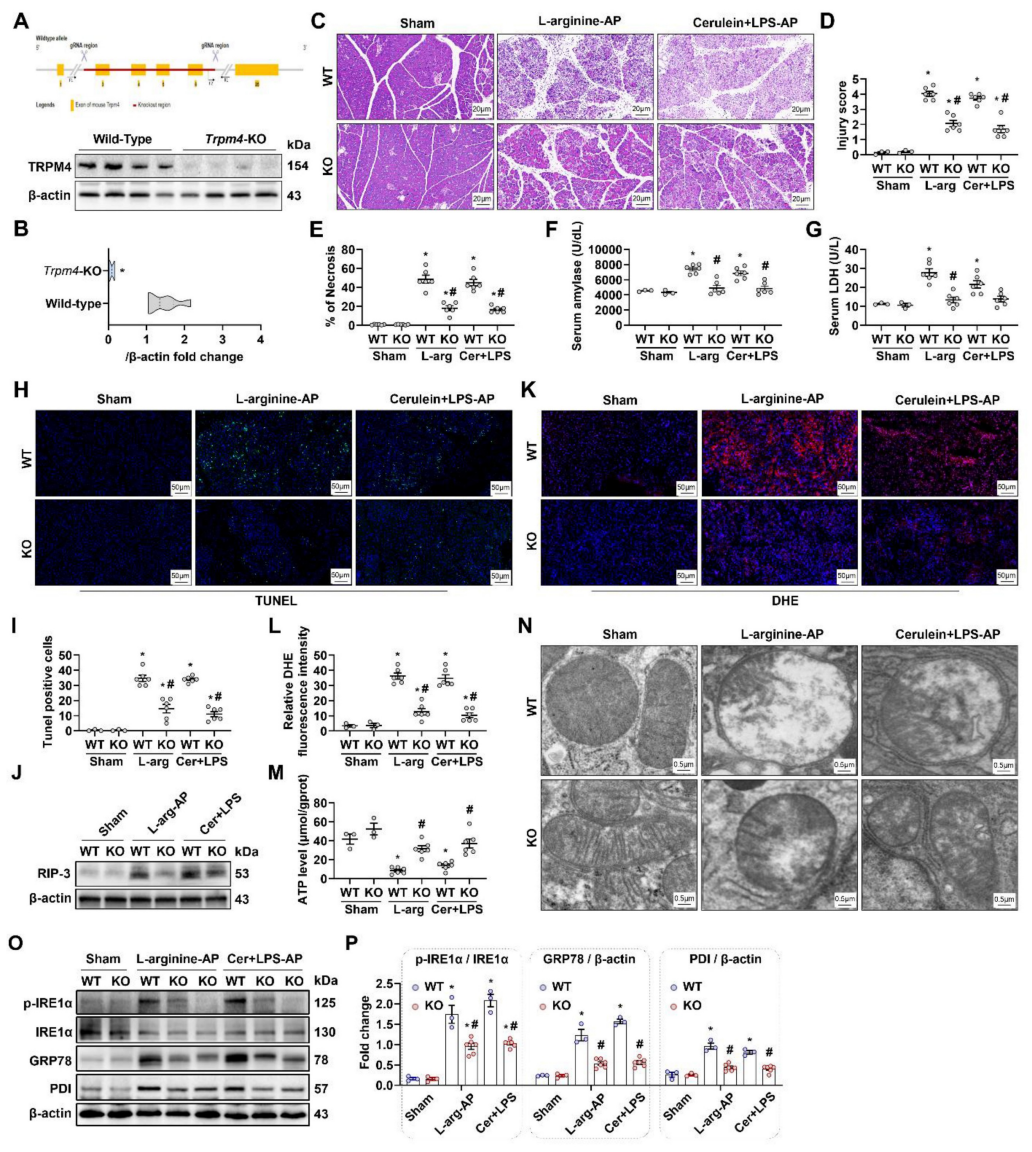
** Pancreatic damage was alleviated in experimental AP after *Trpm4*-knockout.** (**A**) Gene editing schematic and western blot analysis of the TRPM4 expression level in the pancreas; (**B**) Quantitatives of the TRPM4 expression level in the pancreas; (**C**) Representative images of H&E staining of the pancreas (200X); (**D**) Pancreatic injury scores; (**E**) Percentages of necrotic areas; (**F**) Serum amylase levels; (**G**) Serum LDH levels; (**H**) Representative images of TUNEL staining (200X); (**I**) Quantitative of TUNEL staining; (**J**) Western blot analysis of the RIP3 expression level in the pancreas; (**K-L**) Representative images and relative fluorescence intensity of DHE staining in the pancreas (600X); (**M**) ATP levels in the pancreas; (**N**) Ultrastructural alterations in the pancreas; (**O-P**) Western blot analysis and quantitatives of the GRP78, phosphor-IRE1α, IRE1α and PDI expression level in the pancreas. n = 3-6, Error bars indicate the SEM; ∗ P < 0.05 vs Sham; # P < 0.05 vs Vehicle. RIP3, receptor-interacting protein kinase 3; LDH, lactate dehydrogenase; TUNEL, TdT-mediated dUTP Nick-End Labeling; AP, acute pancreatitis; DHE, Dihydroethidium; GRP78, glucose-regulated protein 78; WT, Wild-type; KO, knockout; LPS, lipopolysaccharide.

*Trpm4* gene knockout (KO) mice were successfully constructed (Figures 4A-B), and we induced AP in this animal to observe the effect of defective TRPM4 expression on AP in mice. As shown in Figures 4C-E, the pancreatic damage in *trpm4*-KO mice was observably alleviated compared with that in wild-type (WT) mice with experimental AP. Additionally, serum amylase and LDH levels decreased to varying degrees in AP mice after *trpm4* KO (P < 0.05, Figures 4F-G). TUNEL fluorescence staining and changes in the RIP-3 expression level confirmed that defective TRPM4 expression alleviated pancreatic injury in AP mice (P < 0.05, Figures 4H-J). Similarly, pancreatic oxidative stress (Figures 4K-L), mitochondrial damage (Figures 4M-N), ER stress (Figures 4O-P) and inflammation (Figure S3) induced by L-arginine or cerulein+ LPS treatment were also alleviated after *trpm4*-KO in mice.

### The overexpression of trpm4 in AR42J aggravated cerulein-induced cell death and mitochondrial dysfunction

To clarify the role of TRPM4 in pancreatic exocrine acinar cell injury in AP, we overexpressed *trpm4* in AR42J cells (rat pancreatic exocrine cells) by plasmid transfection. As shown in Figure S4, western blot analysis revealed that the transfection of *trpm4* (Pl-*Trpm4*) led to an increase in the expression of TRPM4 protein in AR42J. We treated AR42J cells with cerulein+ LPS to mimic pancreatic cell injury in AP *in vitro*.

**Figure 5:**
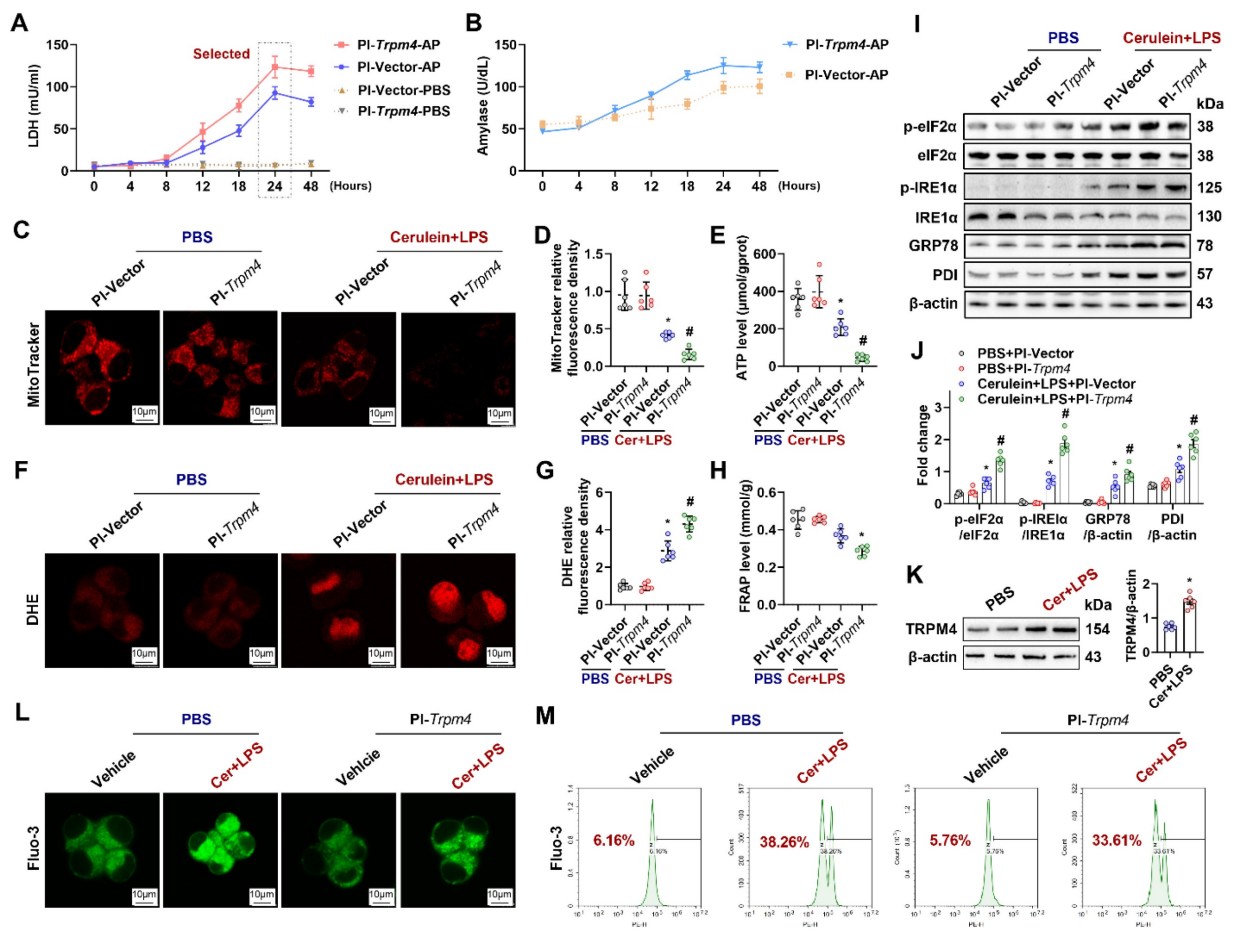
** Overexpression of TRPM4 in AR42J aggravated cerulein induced cell death and mitochondrial dysfunction.** (**A**) Supernatant LDH levels; (**B**) Supernatant amylase levels; (**C-D**) Representative images and relative fluorescence intensity of Mito-Tracker red (1500X) in AR42J cells; (**E**) ATP levels in AR42J; (**F-G**) Representative images and relative fluorescence intensity of DHE (1500X) in AR42J cells; (**H**) FRAP levels in AR42J; (**I-J**) Western blot analysis and quantitative of the phosphor-eIF2α, eIF2α, phosphor-IRE1α, IRE1α, GRP78 and PDI expression level in AR42J; (**K**) Western blot analysis and quantitative of the TRPM4 expression level in AR42J; (**L**) Representative images of immunofluorescence staining of Fluo-3 (1500X) in AR42J cells; (**M**) Flow cytometry analysis of Fluo-3 in AR42J cells. n = 6, error bars indicate the SEM; ∗ P < 0.05 vs Sham; # P < 0.05 vs Vehicle. DHE, Dihydroethidium; LPS, lipopolysaccharide; TRPM4, Transient receptor potential cation channel melastatin 4; GRP78, glucose-regulated protein 78; LDH, lactate dehydrogenase; 9-phen, 9-phenanthrol; Cer, Cerulein; FRAP, Ferric ion reducing antioxidant power; Pl, plasmid.

As shown in Figure 5A, within 24 h after cerulein+ LPS treatment, LDH levels in the supernatant of Pl-Vector treated AR42J (control group) gradually increased (Blue line). From 24 h to 48 h, the level of LDH in the supernatant fluctuated slightly, but the level at 48 h was not significantly different from that at 24 h (P > 0.05). Transfection with Pl-*trpm4* caused cells to release more LDH at 24 h after cerulein+ LPS treatment than that in control group (Pink line, P < 0.05, Figure 5A). An analogical phenomenon was observed for amylase levels in the cell supernatant (Figure 5B), suggesting that the overexpression of TRPM4 aggravated the destruction of AR42J in the *in vitro* AP model.

The Mito-Tracker fluorescent dye stains mitochondria in living cells, reflecting the number of functioning mitochondria 24. As shown in Figures 5C-E, cerulein+ LPS decreased Mito-Tracker fluorescence density and ATP levels in cultured AR42J cells. The overexpression of TRPM4 further decreased Mito-Tracker fluorescence intensity and ATP levels in cerulein+ LPS-treated AR42J cells (P < 0.05). DHE staining and FRAP showed that oxidative stress occurred in the *in vitro* AP models and that oxidative stress was further exacerbated by the overexpression of TRPM4 (Figures 5F-H). The expression of UPR-related proteins showed that ER stress, which occurred in the *in vitro* AP model, was also exacerbated by TRPM4 overexpression (P < 0.05, Figures 5I-J).

Mitochondrial damage and cell death during AP is mainly Ca^2+^-overload dependent 10. Elevated cytoplasmic Ca^2+^ concentrations in the cytoplasm also activate TRPM4 17. In this study, we found that both the expression level of TRPM4 and the concentration of cytoplasmic Ca^2+^ (Fluo-3 labeled green fluorescence) increased in the *in vitro* AP model (Figures 5K-L). The overexpression of TRPM4 aggravated cerulein+ LPS-induced damage to AR42J but had no significant effect on the concentration of Ca^2+^ in the cytoplasm (Figure 5L). Flow cytometry confirmed this phenomenon, and there was no significant difference in Ca^2+^ concentration between AR42J^PBS^ and AR42J^PI-*Trpm4*^ cells before or after cerulein+ LPS treatment (Figure 5L-M). These evidences suggest that there may be an entangled relationship between TRPM4 and Ca^2+^ in the occurrence of mitochondrial damage in AP.

### 9-Phenanthrol inhibited cerulein-induced cell death and mitochondrial damage in AR42J cells

**Figure 6:**
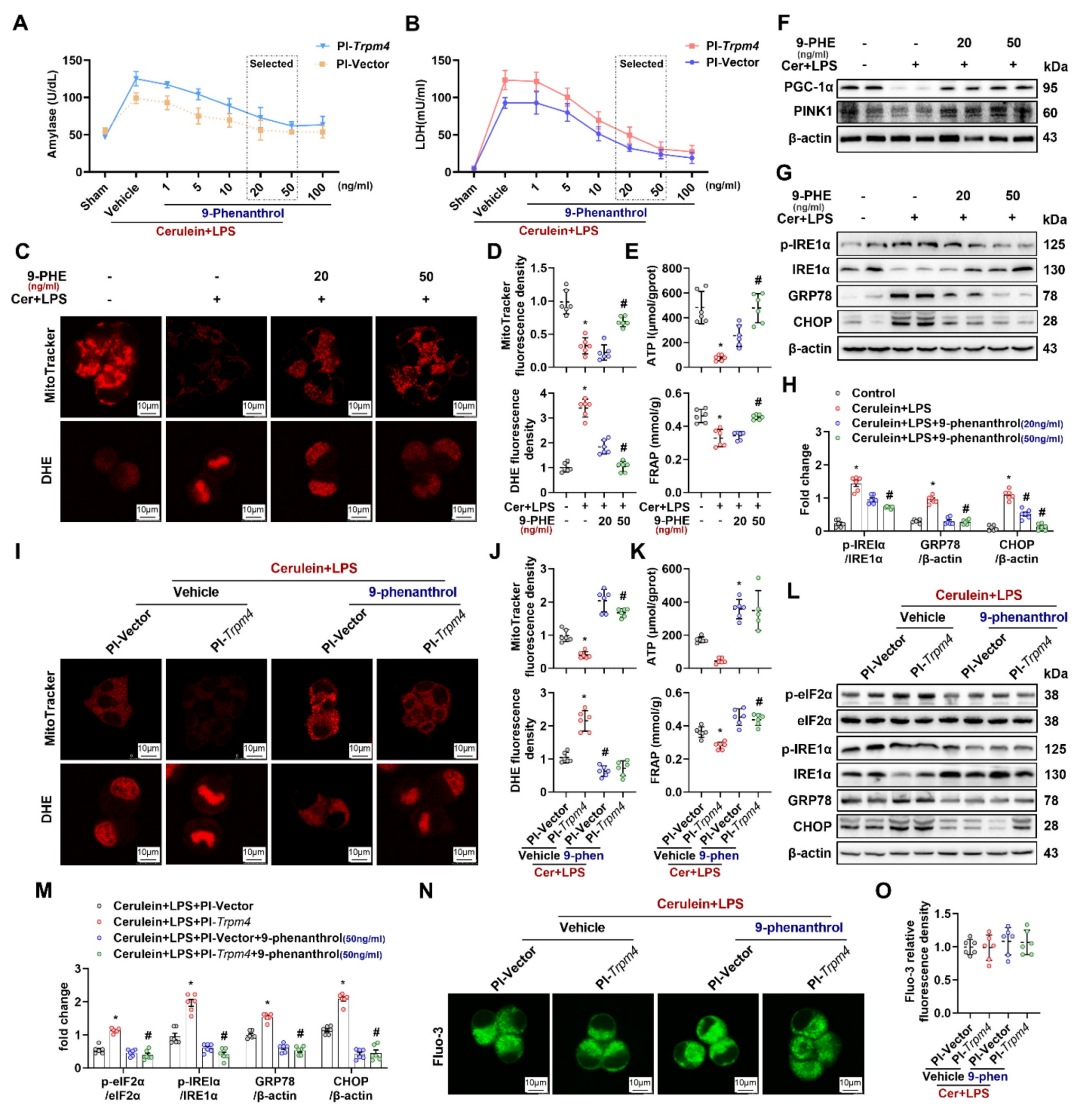
** 9-phenanthrol inhibited cerulein induced cell death and mitochondrial dysfunction.** (**A**) Supernatant amylase levels; (**B**) Supernatant LDH levels; (**C**) Representative images of Mito-Tracker red and DHE (1500X) in AR42J cells; (**D**) Relative fluorescence intensity of Mito-Tracker red and DHE in AR42J cells; (**E**) ATP levels and FRAP levels in AR42J; (**F**) Western blot analysis of the PGC-1α and PINK1 expression level in AR42J; (**G-H**) Western blot analysis and quantitative of the phosphor-IRE1α, IRE1α, GRP78 and CHOP expression level in AR42J; (**I**) Representative images of Mito-Tracker red and DHE (1500X) in AR42J cells; (**J**) Relative fluorescence intensity of Mito-Tracker red and DHE in AR42J cells; (**K**) ATP levels and FRAP levels in AR42J; (**L-M**) Western blot analysis of the phosphor-eIF2α, eIF2α, phosphor-IRE1α, IRE1α, GRP78 and CHOP expression level in AR42J; (**N-O**) Representative images and relative fluorescence intensity of Fluo-3 (1500X) in AR42J cells. n = 3-4, error bars indicate the SEM; ∗ P < 0.05 vs Sham; # P < 0.05 vs Vehicle. DHE, Dihydroethidium; LPS, lipopolysaccharide; GRP78, glucose-regulated protein 78; LDH, lactate dehydrogenase; CHOP, C/EBP homologous protein; FRAP, Ferric ion reducing antioxidant power; Pl, plasmid.

To verify the direct protective effect of TRPM4 inhibition on AR42J, we added different doses of 9-phenanthrol to cerulein+ LPS-treated AR42J cells at 24 h (Pl-*trpm4* or Pl-*vector*). As shown in Figures 6A-B, with the increase of 9-phenanthrol, the level of amylase and LDH in the supernatant of vehicle- or PI-*Trpm4*- treated AR42J cells gradually decreased. The lowest level was observed in cells treated with 50 ng/ ml and 100 ng/ ml 9-phenanthrol (P < 0.05). Mito-Tracker and DHE fluorescence staining showed that 9-phenanthrol relieved cerulein+ LPS induced mitochondrial damage and oxidative stress in AR42J cells (Figures 6C-E). 9-phenanthrol also directly restored mitochondrial PGC-1α and PINK1 expression (Figure 6F), alleviated ER stress in cerulein+ LPS treated AR42J (Figures 6G-H). The expression level of CHOP increases during ER stress, and CHOP mediating ER stress- related programmed cell death in AP 25. In this study, 9-phenanthrol decreased the level of CHOP. Figures 6I-M showed that 50 ng/ ml 9-phenanthrol alleviated the aggravation of mitochondrial damage and ER stress caused by TRPM4 overexpression in cerulein+ LPS- treated AR42J.

Quantification of Fluo-3 fluorescence density showed that the TRPM4 inhibitor had no significant effect on the concentration of Ca^2+^ in the cytoplasm of the *in vitro* AP models (P > 0.05, Figures 6N-O). These results, combined with those in Figures 5L-M, showed that altering TRPM4 levels in AR42J cells affect mitochondrial dysfunction and cell death without affecting intracellular Ca^2+^ concentration. It is speculated that intracellular Ca^2+^ overload possibly induce mitochondrial dysfunction and AP through TRPM4 mediation. To further elucidate this perspective, we applied Yoda1 to overload Ca^2+^ in mouse pancreatic acinar cells (PACs). Yoda1 is an effective agonist of Piezo1, the activation of which leads to Ca^2+^ overload in acinar cells and induces AP 26.

As illustrated in Figure S5A, the Ca^2+^ concentration in Yoda1-treated mouse PACs increased rapidly, and over time, the levels of LDH in the cell supernatant also gradually increased (P < 0.05, Figure S5B). Consistent with our conjecture, the expression level of TRPM4 increased with the continuous increase of intracellular Ca^2+^ (Figure S5C). And the change trend of TRPM4 expression was similar to that of LDH level in cell supernatant. 9-phenanthrol alleviated the aforementioned damage in a dose-dependent manner (Figure S5D) and restored the mitochondrial capacity for ATP production and antioxidation (Figures S5E-F), without affecting Yoda1-induced Ca^2+^ level increase (Figure S5G). These results provide evidence that TRPM4 serves as a mediator in the process by which Ca^2+^ overload disrupts mitochondrial function in acinar cells and promotes cell death.

### NMDARs interacts with TRPM4 to induce ER stress and cell death in AR42J

**Figure 7:**
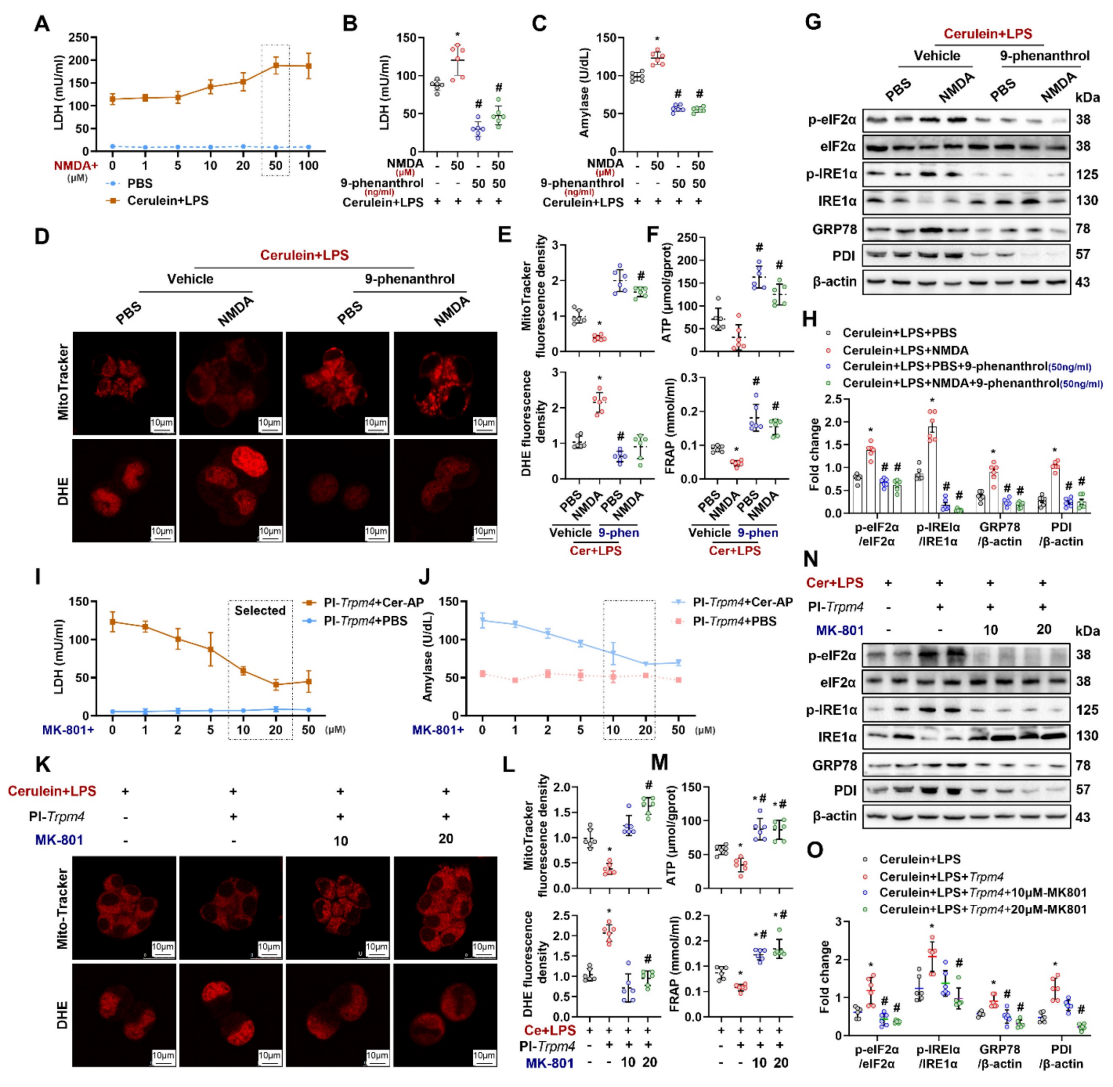
**NMDAR interacts with TRPM4 to induce ER stress and cell death in AR42J.** (**A-B**) Supernatant LDH levels; (**C**) Supernatant amylase levels; (**D**) Representative images of Mito-Tracker red and DHE (1500X) in AR42J cells; (**E**) Relative fluorescence intensity of Mito-Tracker red and DHE in AR42J cells; (**F**) ATP levels and FRAP levels in AR42J; (**G-H**) Western blot analysis and quantitative of the phosphor-eIF2α, eIF2α, phosphor-IRE1α, IRE1α, GRP78 and PDI expression level in AR42J; (**I**) Supernatant LDH levels; (**J**) Supernatant amylase levels; (**K**) Representative images of Mito-Tracker red and DHE (1500X) in AR42J cells; (**L**) Relative fluorescence intensity of Mito-Tracker red and DHE in AR42J cells; (**M**) ATP levels and FRAP levels in AR42J; (**N-O**) Western blot analysis and quantitative of the phosphor-eIF2α, eIF2α, phosphor-IRE1α, IRE1α, GRP78 and PDI expression levee in AR42J. n = 6, error bars indicate the SEM; ∗ P < 0.05 vs Sham; # P < 0.05 vs Vehicle. DHE, Dihydroethidium; LPS, lipopolysaccharide; GRP78, glucose-regulated protein 78; LDH, lactate dehydrogenase; FRAP, Ferric ion reducing antioxidant power; Pl, plasmid; NMDA: N-methyl-d-aspartate.

The interaction between NMDARs and TRPM4 plays a momentous role in mitochondrial damage in neurons 18. To explore the mechanism by which TRPM4 affects pancreatic cells damage, an NMDARs agonist was added to the *in vitro* AP model. As shown in Figure 7A and Figure S6, after the addition of 50 μM NMDARs agonist (NMDA+), the levels of LDH and amylase in the supernatant of cerulein+ LPS-treated AR42J was increased (P < 0.05). 9-Phenanthrol (50 ng/ ml) inhibited the increase in LDH and amylase levels induced by NMDA+ (Figures 7B-C). Analogously, the NMDA+-induced worsening of mitochondrial damage and ER stress in the *in vitro* AP model was attenuated by the administration of 9-phenanthrol (Figures 7D-H). These results imply that the cellular dysfunction induced by NMDARs activation during AP is dependent on the presence of TRPM4.

To test the aforementioned hypothesis, we introduced MK-801+, an NMDARs inhibitor, into the *in vitro* AP model. *Trpm4*-overexpressing AR42J were treated with different concentrations of MK-801 plus cerulein + LPS. As shown in Figures 7I-J, MK-801 at different concentrations had no significant effect on PBS-treated TRPM4-overexpressing AR42J cells. However, with the increase in MK-801 concentration, the aggravation of cell damage caused by TRPM4 overexpression in cerulein+ LPS- treated AR42J cells was gradually alleviated. MK-801 (20 μM) significantly reduced LDH and amylase levels in the supernatant of AR42J cells (P < 0.05). The same protective effect of MK-801 was also observed in mitochondrial and ER stress in AR42J^PI-*Trpm4*^ cells treated with cerulein+ LPS (Figures 7K-O).

**Figure 8:**
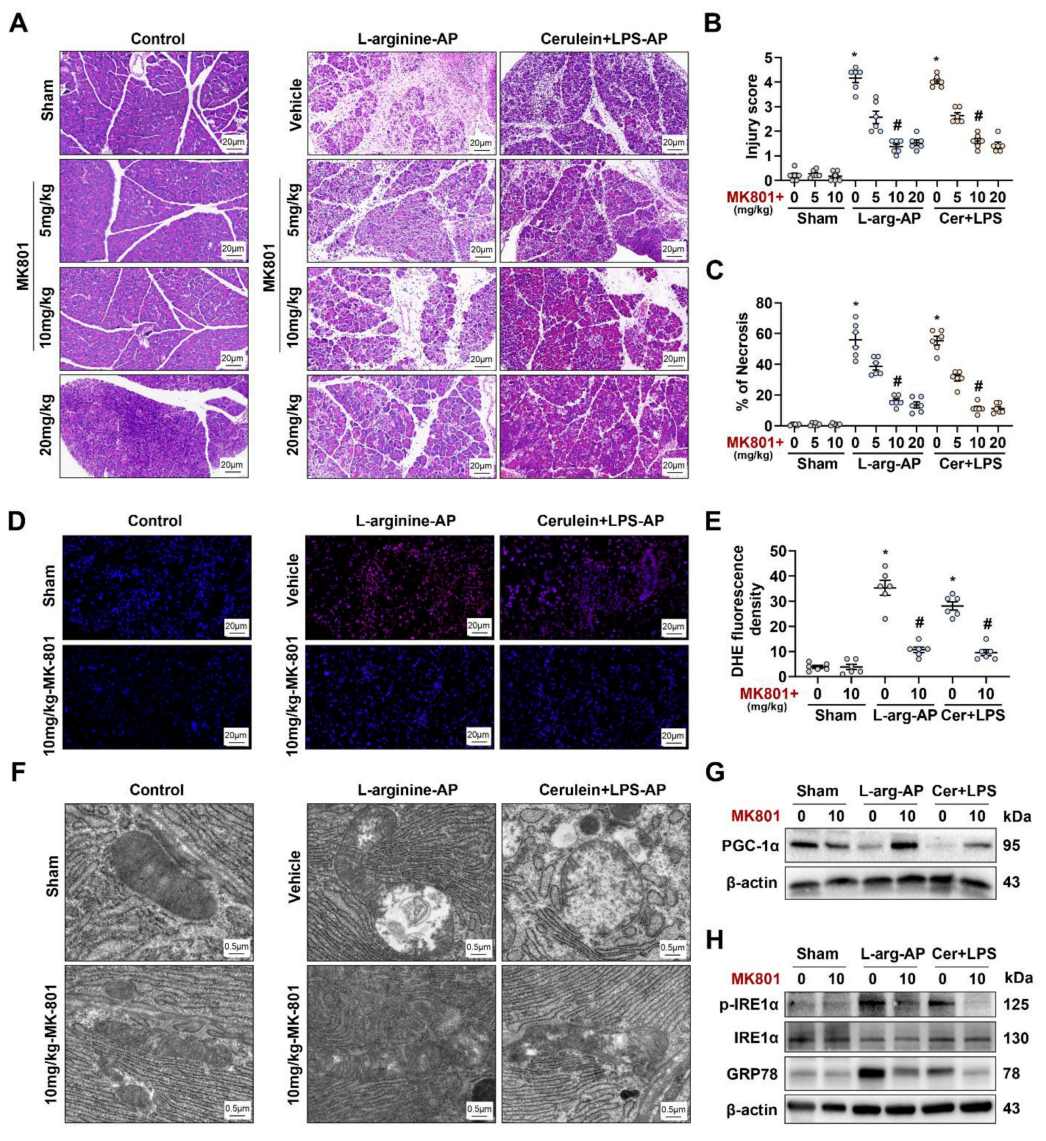
**NMDAR interacts with TRPM4 to induce ER stress and pancreatic injury in experimental AP.** (**A**) Representative photos of H&E staining of the pancreas (200X); (**B**) Pancreatic injury scores; (**C**) Percentages of necrotic areas; (**D-E**) Representative images and relative fluorescence intensity of DHE staining in the pancreas (400X); (**F**) Ultrastructural alterations in the pancreas; (**G**) Western blot analysis of the PGC-1α expression level in the pancreas; (**H**) Western blot analysis of the GRP78, phosphor-IRE1α and IRE1α expression level in the pancreas. n = 3-6, error bars indicate the SEM; ∗ P < 0.05 vs Sham; # P < 0.05 vs Vehicle. AP, acute pancreatitis; GRP78, glucose-regulated protein 78; LPS, lipopolysaccharide.

We applied MK801 to* in vivo* AP models to validate the results obtained *in vitro*. The intraperitoneal injection of different doses of MK801 alleviated pancreatic damage caused by L-arginine or cerulein + LPS (Figures 8A-C) without affecting TRPM4 expression levels in the pancreas of AP model (Figures S7A-B). In addition, we observed a significant change at a dose of 10 mg/kg MK801 (P < 0.05, Figures 8A-C). Similar to the results observed *in vitro*, MK801 alleviated ROS deposition, mitochondrial spoilage and ER stress in AP mice (Figures 8D-H). This evidence confirms that NMDARs interacts with TRPM4 to induce intracellular dysfunction and cell death in AP.

## Discussion

**Figure 9:**
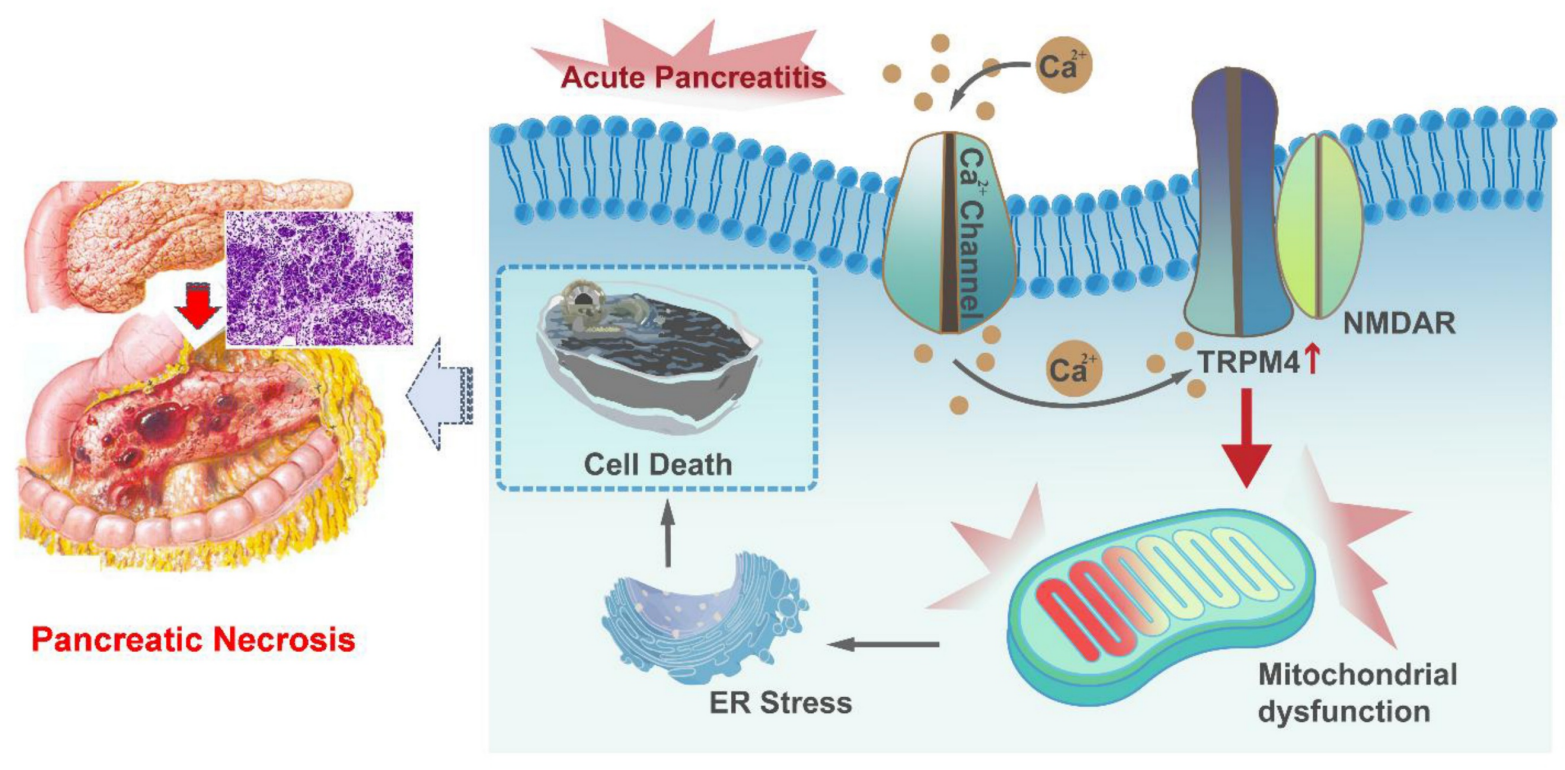
**Graphical abstract.** When AP occurs, Ca^2+^ overload leads to ER stress and cell death through TRPM4/NMDARs-mediated mitochondrial dysfunction in pancreatic exocrine acinar cells.

Herein, we demonstrated that the expression level of TRPM4 is increased in the pancreatic tissues of AP mice. We used three animal models of AP, with the aim of conduct preclinical studies that mimic the different pathogeneses of human disease. The study proved that intraperitoneal injection of TRPM4 inhibitors had a protective function on pancreatic injury in various AP model. *In vitro* experiments confirmed that the damage to pancreatic exocrine acinar cells caused by TRPM4 activation was dependent on the presence of NMDARs. TRPM4, in conjunction with functional NMDARs, mediates mitochondrial dysfunction in acinar cells and exacerbates intracellular oxygen free radical accumulation and ER stress, ultimately leading to cell death (Figure 9).

TRPM4 is a member of the transient receptor potential (TRP) channel family. Previous studies have revealed that the TRPM subfamily is associated with neurodegeneration 27. TRPM4 is a Ca^2+^-impermeable cationic channel protein that can be activated by intracellular calcium, depolarization, and variation in temperature 28. TRPM4 is known to be involved in neuropathy, cardiac rhythmopathies, and tumor-related diseases by disrupting mitochondrial homeostasis 15, 29; however, its role in AP has not been reported. Currently, it is believed that the key pathogenic mechanism of AP is pancreatic acinar cell mitochondrial damage caused by Ca^2+^ overload 10, 30. As mentioned above, intracellular Ca^2+^ is involved in the activation of TRPM4, and TRPM4, in turn, is associated with abnormal mitochondrial function. Therefore, it is reasonable to speculate that Ca^2+^ overload in acinar cells caused by different reasons activates TRPM4 and may be participated in the pathogenesis of AP. In this study, both Ca^2+^ and TRPM4 were elevated in AP models. However, the overexpression of *trpm4* in pancreatic acinar cells and the application of inhibitors to block TRPM4 both affected mitochondrial metabolism without affecting the intracellular Ca^2+^ overload. Changes in TRPM4 appear to be a downstream factor affecting cell death that should result from high intracellular Ca^2+^ concentration in AP. These findings manifest that elevated Ca^2+^ in the cytoplasm of pancreatic exocrine acinar cells during AP may affect mitochondrial function by activating TRPM4.

Ca^2+^ overload in acinar cells of AP results from multiple factors. Impaired endoplasmic reticulum Ca^2+^ handling and abnormal activation of plasma membrane ion channels, triggered by inflammation and oxidative stress, are key contributors 10. The role of NMDA receptors in this process is emerging but not fully clear. Pancreatic injury may release glutamate, activating NMDA receptors on acinar cells and promoting Ca^2+^ influx 31. NMDA receptor activation could also dysregulate other Ca^2+^ - handling proteins, worsening Ca^2+^ dyshomeostasis 32. However, the involvement of other glutamate receptor subtypes and interactions with other Ca^2+^ - regulating pathways complicate the picture. Future research using selective NMDA receptor antagonists or genetic models is needed to precisely define NMDA receptors' contribution to Ca^2+^ overload, which could reveal new therapeutic targets for AP.

Members of the TRPM family are not only Ca^2+^-activated cation receptors but also sense temperature changes and produce corresponding functional changes 33. Our recent work showed that the expression of cold-inducible RNA-binding protein (CIRBP), a temperature-sensitive protein, was increased in AP. Inhibiting the expression of extracellular CIRBP by different means helps to decrease the severity of AP 34. Therefore, we wondered whether the aggravation of AP induced by CIRBP was also achieved through TRPM4 activation. Because of this low-temperature dependent activation property of TRPM4, it may be a key target for exogenous CIRBP-induced mitochondrial dysfunction of pancreatic acinar cells. Of course, this hypothesis remains to be explored further.

NMDA receptors (NMDARs), a subtype of ionogenic glutamate receptors, are heteromers composed of multiple subunits and are mainly distributed in the central nervous system 35. Recent evidence shows that NMDARs are not only widely distributed in tissues such as kidney, pancreas, liver and blood vessels but also participate in the maintenance of normal physiological functions of these organs and tissues 36-40. TRPM4 can physically interact with NMDARs and form the NMDARs/TRPM4 complex, which is involved in mediating neuronal mitochondrial disorder, leading to cell death 18. Han et al. also found that the NMDARs inhibitor MK801 suppressed LPS-induced mitochondrial damage and apoptosis in endothelial cells 41, suggesting that the presence of NMDARs is involved in the regulation of cellular mitochondrial homeostasis and participated in the destruction of cells caused by damaging factors. In this study, we showed that the activation of TRPM4 resulted in abnormal mitochondrial function and increased cell death in AP. This effect of TRPM4 also appears to be NMDARs dependent. As the NMDARs inhibitor MK-801 inhibited mitochondrial damage aggravated by *trpm4* overexpression in the *in vitro* AP model. Furthermore, 9-phenanthrol, an inhibitor of TRPM4, rescues mitochondrial function and cell death exacerbated by NMDARs agonists, indicating that both functional TRPM4 and NMDARs in pancreatic exocrine acinar cells is a key link mediating mitochondrial dysfunction during AP.

Moreover, we propose that the interplay between NMDA receptors and TRPM4 channels may involve both physical interactions mediated by proteins like TwinF (as reported by Yan et al.) and functional coupling, where Ca²⁺ influx via NMDA receptors directly activates TRPM4 through Ca²⁺/calmodulin-dependent mechanisms in AP. This dual-mode interaction, combining structural association and Ca²⁺-dependent functional regulation, could synergistically modulate cellular excitability and the function of downstream mitochondria. However, due to the lack of evidence, further studies (e.g., Co-IP assays, Ca²⁺-binding site mutagenesis and other functional experiments) are needed to dissect their respective contributions.

Mitochondria are the "energy factories" of eukaryotic cells and perform oxidative reactions and produce ATP. The stability of their function and morphology maintains the homeostasis of cells 19, 42. Mitochondrial dysfunction is a key factor in exocrine cells damage in AP 10, 43. Abnormal mitochondrial function, such as impaired mitochondrial selective autophagy (otherwise known as mitophagy), reduces the oxidative metabolic capacity of a cell, leading to the accumulation of oxygen free radical products 44 and cause oxidative stress 45. Non-hypoxic induction of mitophagy can be modulated by one of the three classical pathways, the PINK1/Parkin pathway, which plays causative roles in neurodegenerative disease 46. In this study, we found that the expression of mitophagy protein PINK1 was down-regulated during AP, indicating that AP caused abnormalities in the catabolic process of autophagosome - lysosome to mitochondrial degradation products, including oxygen free radical products. The imbalance of redox process in the cell in turn increases the burden of mitochondria, and then a series of organelle dysfunction occurs. At present, it has been confirmed that ferroptosis is often caused by abnormal mitophagy and the oxidative stress. The combination of these injury factors resulted in irreversible damage of acinar cells during AP, which aggravated tissue destruction 47, 48. We have demonstrated in both *in vivo* and *in vitro* that inhibition of TRPM4 restores the expression of mitophagy protein PINK1, suggesting that TRPM4-mediated mitochondrial dysfunction is partly caused by affecting its normal autophagy process.

As "energy factories", the decrease in energy production capacity of mitochondria further affects the function of other organelles in the cell, induces ER stress, and reduces the ability of the ER to properly fold proteins. These abnormal functions promote the occurrence of impaired intracellular autophagy (otherwise known as macrophagy), aggravate lipid metabolism disorders in acinar cells, give rise to the expression of apoptosis-related proteins, and ultimately lead to cell death 10, 11. TRPM4 is involved in mitochondrial damage and oxidative stress in cardiomyocytes 49, its role in other organelles injury of AP has not been discussed. In this study, the application of 9-phenanthrol to antagonize TRPM4 alleviated the increased mitochondrial damage in both* in vivo* and *in vitro* AP models. And this protective effect of 9-phenanthrol appears to be generated by restoring normal mitochondrial biogenesis and fusion, such as the restoration of PGC-1α and Mfn-2 expression. By saving mitochondrial functional proteins, the core function of mitochondria - the ability to produce ATP for cells - was also improved. And then, the high expression of the ER stress-related protein CHOP, which mediates apoptosis, decreased after TRPM4 inhibition. Glucose-regulated protein 78 (GRP78) is a hallmark chaperone induced by ER stress. It is vital for protein folding and assembly in the ER and regulation of ER stress initiation mediators, was also decreased after TRPM4 inhibition. The saved endoplasmic reticulum retains the ability to process proteins, promoting the rehabilitation of cell function. The remission of necrosis and inflammation showed that 9-phenanthrol not only restored cell function at the microscopic level but also reduced the damage caused by AP at the macroscopic level. With reference to previous studies on mitochondrial damage in AP, we suggest that these general improvements should be secondary to the protection of mitochondrial function.

There are some limitations to the study. Herein, we discussed the possible role of the NMDARs/TRPM4 complex in acinar cell damage during AP and proposed that the mitochondrial damage in acinar cells caused by intracellular Ca^2+^ overload may be mediated by the NMDAR/TRPM4 complex. However, the specific downstream molecules of NMDAR/TRPM4 that regulate mitochondrial function in exocrine cells during AP is still unknown. The ABCE1 gene is a member of the ATP binding box transporter gene subfamily, and the regulation of ABCE1 alleviates impaired mitochondrial function by affecting mitophagy 50. In this study, we observed changes in ABCE1 expression when NMDARs/TRPM4 was modulated to affect mitochondrial function (unpublished data). This phenomenon manifests that ABCE1 may be implicated in the regulation of mitochondrial function by NMDAR/TRPM4. Therefore, our future research goals are to explore the role of ABCE1 in mitochondrial damage in pancreatic cells, confirm whether ABCE1 mediates the NMDAR/TRPM4-induced disruption of mitochondrial function during AP, and elucidate the possible molecular signaling pathway through which NMDAR/TRPM4 regulates ABCE1, thereby regulating mitophagy and ultimately affecting mitochondrial function in AP.

The activation of TRPM4 leading to mitochondrial dysfunction may also be achieved by affecting the mitochondrial membrane potential (ΔΨm). As mentioned before, TRPM4 is a Ca^2+^ -activated cation channel that alters the cell membrane potential by regulating the flow direction of cations. Plasma membrane potential changes may directly interfere with mitochondrial electrochemical gradients via voltage-dependent ion channel crosstalk. Our experiments shows that the expression of ABCE1 changes under the above circumstances, but its role in regulating ΔΨm remains unvalidated. The potential contribution of TRPM4-induced ΔΨm disruption warrants mechanistic exploration, particularly regarding ABCE1-mediated trafficking or functional coupling.

Another limitation of this study is regarding the application of inhibitors. While 9-phenanthrol was used here based on prior research, its potential off-target effects highlight a limitation, as 4-chloro-2-[2-(2-chloro-phenoxy)-acetylamino]-benzoic acid (CBA) shows more selectivity for TRPM4 and meclofenamate has been validated in animal models 17, 51. Future studies using these selective inhibitors will help dissociate TRPM4-specific effects from off-target actions, strengthening mechanistic insights into TRPM4-mediated mitochondrial damage.

In conclusion, the results of this study prove that TRPM4 expression in acinar cells mediates mitochondrial damage during AP, contributing to organelle dysfunction and cell death. This involvement of TRPM4 in acinar cell damage may be NMDARs dependent. In addition, the NMDAR/TRPM4 complex may be a key signaling node in intracellular Ca^2+^ overload-induced mitochondrial damage and AP.

## Materials and Methods

***Animals:*** Male C57BL/6 J mice (age, 9-10 weeks; weight, 20-22 g) were purchased from Xi 'an Huaren Biotechnology Co., LTD and housed in the Experimental Animal Center of Xi'an Jiaotong University. *Trpm4* gene knockout (*trpm4*-KO) mice (Cyagen Biosciences, Inc., Jiangsu, CN) were generated and used in this study, Detailed primer sequences are listed in the supplementary material. Wild-type littermates were used as controls.

***In vivo* models:** Mice were fasted for 8-12 h before the procedure. Arginine-induced AP mice were generated by 2 hourly intraperitoneal injections of 4 g/kg L-arginine (A640158, Aladdin Scientific, CN) 12. The animals were anesthetized by isoflurane inhalation at 24, 48 or 72 h after the first injection of L-arginine. Blood samples and pancreatic tissues were collected. In other groups of L-arginine-induced AP model, at 3 h after the first injection of L-arginine, normal saline (vehicle) or 20, 50, 100 μg/kg 9-phenanthrol (E0028, Selleck, Inc. CN) was administered via intraperitoneal injection. The animals were anesthetized at 72 h after the first injection of L-arginine (i.e., 69 h after 9-phenanthrol treatment).

At 3 h after the first injection of L-arginine, normal saline (vehicle) or 5, 10, 20 mg/kg MK-801 (S2876, Selleck, Inc. CN), a specific NMDA receptor antagonist, was administered by intraperitoneal injection. The animals were anesthetized at 72 h after the first injection of L-arginine (i.e., 69 h after MK-801 treatment). In additional groups of L-arginine-induced AP, survival was monitored for 5 days after vehicle treatment or 9-phenanthrol.

Cerulein + LPS-induced AP mice were generated by 7 hourly intraperitoneal injections of 50 μg/kg cerulein (C6660, Solarbio, CN). 10 mg/kg lipopolysaccharide (LPS) (L8880, Solarbio, CN) was added to the last cerulein injection 13. The animals were anesthetized at 8, 11 or 15 h after the first injection of cerulein (i.e., 1, 4 or 8 h after the last injection of cerulein). In another group of cerulein + LPS-induced AP mice, at 30 min after the second injection of cerulein, normal saline (vehicle) or 20, 50, 100 μg/kg 9-phenanthrol was administered. The animals were sacrificed at 11 h after the first injection of cerulein.

At 30 min after the second injection of cerulein, normal saline (vehicle) or 5, 10, 20 mg/kg MK-801 (S2876, Selleck, Inc. CN), a specific NMDA receptor antagonist, was administered via intraperitoneal injection. The animals were sacrificed at 11 h after the first injection of cerulein. In additional groups of cerulein + LPS-induced AP, survival was monitored for 5 days after vehicle treatment or 9-phenanthrol.

Cerulein-induced AP mice were generated by 7 hourly intraperitoneal injections of 50 μg/kg cerulein (C6660, Solarbio, CN). The animals were anesthetized and sacrificed at 8, 11 or 15 h after the first injection of cerulein. In another group of cerulein-induced AP mice, at 30 min after the second injection of cerulein, 50 or 100 μg/kg 9-phenanthrol was administered. The animals were sacrificed at 11 h after the first injection of cerulein. In additional groups of cerulein-induced AP, survival was monitored for 5 days after vehicle treatment or 9-phenanthrol.

**Cell culture:** Pancreatic AR42J cells (CL-0025, Procell, CN) were cultured in AR42J cell specific medium (CM-0025, Procell, CN) in a humidified incubator at 37 °C with 5% CO_2_ 13. Mouse pancreatic acinar cells (PACs) (CP-M226, Procell, CN) were cultured in their specific culture medium (CM-M226, Procell, CN) within the same conditions. Both cells were implanted into six-well culture plates (5×10^5^/well) or laser confocal plates (5×10^5^/well) for subsequent experiments. Before the experiment, pretreatment with 100 nM dexamethasone (D4902, Sigma-Aldrich, Germany) for 48 h was used to activate AR42J cells to differentiate into acinar-like phenotypes.

***In vitro* models in AR42J cells and plasmid transfection:** AR42J cells were treated with cerulein (100 nmol/L, C6660, Solarbio, CN) and LPS (10 ng/ ml, L-8880, Solarbio, CN) for 4, 8, 12, 18, 24 or 48 h. An equal volume of medium was given as a control. A TRPM4-overexpressing plasmid (Pl-*trpm4*) and negative control plasmid (Pl-vector) were synthesized by Shanghai Genechem Corporation (CN). The plasmid was extracted and transfected according to the manufacturer's instructions.

In another experiment, AR42J cells were treated with 100 nmol/L cerulein (C6660, Solarbio, USA) and 10 ng/ ml LPS (L-8880, Solarbio, CN) with or without, 1, 5, 10, 20 50 or 100 ng/ ml 9-phenanthrol (E0028, Selleck, Inc. CN) for 24 h. Pl-*trpm4* and Pl-vector were transfected into the cells following the manufacturer's instructions.

In another experiment, AR42J were incubated in different concentrations of NMDA (1, 5, 10, 20 50 or 100 μM) (S7072, Selleck, Inc. CN), a specific NMDA receptor agonist, or MK-801 (1, 2, 5, 10 20 or 50 μM) (S2876, Selleck, Inc. CN), a specific NMDA receptor antagonist, with or without 50 ng/ ml 9-phenanthrol. Pl-*trpm4* was transfected into the cells following the manufacturer's instructions.

***In vitro* models in mouse PACs cells:** Mouse PACs were re-cultured in Hanks' Balanced Salt Solution (with Ca^2+^ & Mg^2+^) (C0219, Beyotime, CN) and treated with Yoda1 (50 μM, S6678, Selleck, CN) for 1, 2, 3, 10, 20 or 30 min 26, 52. In another experiment, mouse PACs were treated with 50 μM Yoda1 with or without 20 or 50 ng/ ml 9-phenanthrol (E0028, Selleck, Inc. CN) for 30 min. An equal volume of medium was given as a control.

**Statistical analysis:** Data were analyzed using GraphPad Prism 10.1.2 Software (San Diego, California, USA) and expressed as means ± standard error (SEM). The t-test or one-way ANOVA and compared using Student Newman Keuls test was used to analyze the differences between groups. Kaplan-Meier curves were used for survival analysis and log-rank testing for difference analysis. A *P*-value < 0.05 represented a significant difference.

**The methods for** H&E, immunohistochemical and immunofluorescence staining (HSP60); TUNEL, dihydroethidium (DHE), Mito-Tracker and Fluo-3 staining; ATP and FRAP analyses; enzyme-linked immunosorbent assays (ELISA); flow cytometry (FCM); biochemical detection; transmission Electron Microscopy (TEM) and western blot analysis **are provided in the Supplementary Material**.

## Supplementary Material

Supplementary materials and methods, figures and table, blot images, report.

## Abbreviations

AP: Acute Pancreatitis
TRPM4: Transient receptor potential cation channel melastatin 4
NMDARs: N-methyl-d-aspartate receptors
ER stress: Endoplasmic Reticulum stress
Pl: plasmid
ATP: adenosine triphosphate
MODS: multiple organ dysfunction syndrome
RIP3: receptor-interacting protein kinase 3
LDH: lactate dehydrogenase
ROS: reactive oxygen species
TUNEL: TdT-mediated dUTP Nick-End Labeling
ELISA: Enzyme-linked immunosorbent assay
UPR: unfolded protein response
HSP60: heat shock protein 60
TEM: transmission electron microscope
PGC-1α: peroxisome proliferative activated receptor-γ coactivator 1α
DHE: Dihydroethidium
CHOP: C/EBP homologous protein
FRAP: Ferric Reducing Antioxidant Power
IRE1α: inositol-requiring enzyme 1 α
GRP78: glucose-regulated protein 78
LPS: lipopolysaccharide
AR42J: cells of the rat exocrine pancreas
CIRBP: Cold-induced RNA-binding protein
WT: Wild type
KO: knockout
PINK1: PTEN induced putative kinase 1
P/S: penicillin/streptomycin
n.s: non-significant
PACs: Pancreatic acinar cells
FCM: flow cytometry
CBA: 4-chloro-2-[2-(2-chloro-phenoxy)-acetylamino]-benzoic acid
