# Supplementary material for: Blocking TRPM4 alleviates pancreatic acinar cell damage via an NMDA receptor-dependent pathway in acute pancreatitis
Source: Theranostics. 2025 Jun 9;15(14):6901–18. doi: 10.7150/thno.116520 (PMC12203808; doi:10.7150/thno.116520)
Supplement: Supplementary file 1 — Supplementary materials and methods, figures and table, blot images, report. [file thnov15p6901s1.pdf]

Supplementary Materials for

Blocking TRPM4 alleviates pancreatic acinar cell damage via an NMDA receptor-dependent pathway in acute pancreatitis

Yifan Ren, Qing Cui, Wuming Liu, Hangcheng Liu, Tao Wang, Hongwei Lu, Yi Lv*, Rongqian Wu*

*Corresponding author: rwu001@mail.xjtu.edu.cn or luyi169@mail.xjtu.edu.cn

This file includes:

Supplementary Materials and Methods

Supplementary Table;

Figure S1 to Figure S7;

Supplementary Figure Legends;

Raw western blot images;

Report of *trpm4* gene knockout mice.

Supplementary Materials and Methods

***Trpm4* knockout mouse and genotyping strategy:** Name: C57BL/6NCya-Trpm4 em1/Cya. Serial Number: KOCMP-68667-Trpm4-B6N-VA. Type: conventional knockout. Primers 1 (Annealing Temperature 60.0 ℃), F1: 5'-AGTCCCTGCTCATTACTCTGGG-3', R1: 5'-GAGAAGGTAAAGAAGCCTGTGC-3', Product size: 838 bp. Primers 2 (Annealing Temperature 60.0℃), F2: 5'-TGGGAACTTGAGGATGAAGTCAG-3', R1: 5'-GAGAAGGTAAAGAAGCCTGTGC-3', Product size: 643 bp. Homozygotes: one band with 838 bp; Heterozygotes: two bands with 838 bp and 643 bp; Wildtype allele: one band with 643 bp.

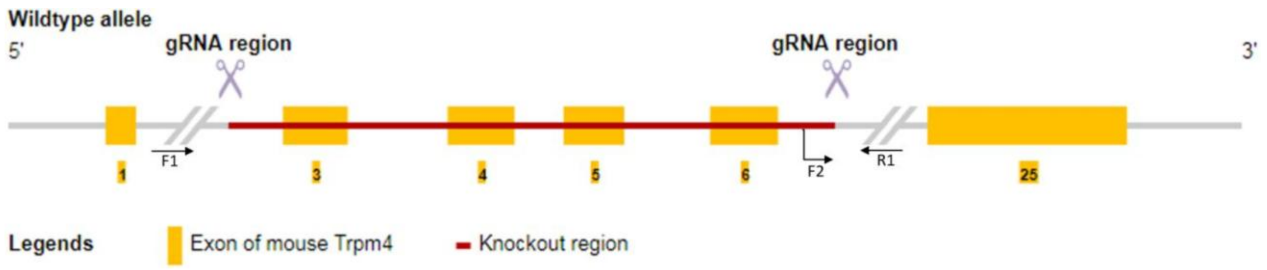

Targeting strategy for trpm4 knockout mouse (Enlarged Figure 4A)

Pathological staining: Pancreas tissue was obtained. The tissue samples were fixed in 10% neutral buffered formalin at room temperature for 24 - 48 h and then rinsed. Next, the fixed tissues undergo dehydration in graded ethanol solutions, clearing in xylene, and infiltration with molten paraffin before being embedded in paraffin blocks. Paraffin - embedded blocks are sectioned to 4 - 5 µm thickness, stretched on a warm water bath, and air - dried on glass slides. The HE staining process involves deparaffinization, rehydration, hematoxylin staining for nuclei, differentiation, blueing, eosin staining for cytoplasm and extracellular matrix, followed by dehydration, clearing, and mounting. The stained sections were digitally scanned with a biopsy scanner (Pannoramic MIDI, 3DHISTECH, Hungary).

Pathological score: After HE - stained pancreatic tissue sections were prepared as described above, a semi -

quantitative histopathological scoring system was employed to evaluate the degree of pancreatic injury. The scoring was performed independently by two experienced pathologists who were blinded to the experimental groups. Acinar cell damage: This was evaluated based on the presence of acinar cell necrosis, vacuolization, and loss of acinar architecture. A score of 0 indicated no damage, 1 for mild damage (less than 25% of acinar cells affected), 3 for moderate damage (25 - 50% of acinar cells affected), and 5 for severe damage (more than 50% of acinar cells affected). Inflammatory cell infiltration: The amount of inflammatory cell infiltration in the pancreatic tissue was assessed. A score of 0 represented no infiltration, 1 for mild infiltration (scattered inflammatory cells), 3 for moderate infiltration (aggregates of inflammatory cells in some areas), and 5 for severe infiltration (diffuse and extensive inflammatory cell infiltration). Edema: The degree of interstitial edema was scored. A score of 0 indicated no edema, 1 for mild edema (slight widening of the interstitium), 3 for moderate edema (obvious separation of tissue structures by fluid), and 5 for severe edema (massive accumulation of fluid with marked tissue distortion).

Water content determination: We assessed the degree of pancreatic edema by evaluating changes in water content. Briefly, pancreatic tissues were dried in a 72 ℃ oven for 2 days. The pancreas water content was calculated as the wet weight/dry weight ratio.

Immunohistochemical staining: 4% formalin-fixed pancreatic tissue was paraffin embedded and sectioned at 4 - 5 µm. After deparaffinization and rehydration, antigen retrieval was done using heat in citrate buffer. Endogenous peroxidase was blocked with 3% hydrogen peroxide, followed by blocking non - specific binding with normal serum. Incubation with primary antibody overnight at 4 °C was followed by a biotinylated secondary antibody and then a streptavidin - peroxidase complex. Chromogenic development with DAB was

followed by hematoxylin counterstaining. Sections were dehydrated, cleared in xylene, and mounted. Immunohistochemical staining was performed and the stained sections were digitally scanned with a biopsy scanner (Pannoramic MIDI, 3DHISTECH, Hungary). The primary antibodies are TRPM4 (ab106200, Abcam, USA) and LY6G (ab238132, Abcam, USA).

ATP content determination: Pancreas tissue or AR42J or mouse PACs homogenate was obtained. Preparation of tissue or cell samples for detection was carried out in accordance with the specifications of the assay kit (S0026, Beyotime, China). As for the detection process, the ATP standard solution is first diluted to the appropriate concentration gradient with the ATP detection lysate. Examples include 0.01, 0.03, 0.1, 0.3, 1, 3, and 10 μM. In subsequent experiments, the concentration range of the standard can be adjusted appropriately according to the concentration of ATP in the sample. Then, the appropriate amount of ATP test solution is prepared in the ratio of 100 μL of ATP test solution required for each sample or standard. Take an appropriate amount of ATP test reagent and dilute it with ATP test reagent diluent at the ratio of 1:4. The diluted ATP detection reagent is the working liquid for ATP detection for subsequent experiments. To determine the ATP content of the sample, first add 100 μL of ATP detection working liquid to the detection tube. Leave at room temperature for 3-5 min, so that all the background ATP is consumed, thereby reducing the background. Then add 20 μL of sample or standard in the test tube, quickly mix with a micropipette, at least 2 s after the luminometer or liquid flash meter to determine the RLU value or CPM. Finally, the concentration of ATP in the sample was calculated according to the standard curve.

Transmission electron microscopy (TEM): Pancreatic tissue was promptly fixed in 2.5% glutaraldehyde in 0.1 M phosphate buffer (pH 7.4) at 4 °C for 2 - 4 h. After rinsing, they were post - fixed with 1% osmium tetroxide.

Dehydration was done using a series of ethanol solutions, followed by infiltration with a mixture of absolute ethanol and epoxy resin, and then embedding in epoxy resin at 60 °C for polymerization. 60-80 nm ultra-thin sections of pancreatic samples were stained with uranyl acetate and lead citrate. Pancreatic ultrastructure, such as mitochondria and endoplasmic reticulum, was evaluated using a transmission electron microscope (HITACHI HT7700, Hitachi, Japan) by a technical microscopist.

TUNEL, HSP60, DHE (Dihydroethidium), MitoTracker and Fluo-3 AM staining: A TUNEL kit (11684795910, Roche, Switzerland), a HSP60 (Antibody, AG2237, Beyotime, China) kit, two DHE (Dihydroethidium for *in vivo*, G1045, Servicebio, China or Dihydroethidium for *in vitro*, S0063, Beyotime, China) kit, a MitoTracker staining kit (Mitochondrial probe, M7512, Thermo Fisher Scientific, Beijing, China) and a Fluo-3 AM staining kit (Calcium ion fluorescent probe, S1056, Beyotime, China) were used for TUNEL, HSP60, DHE, MitoTracker and Fluo-3 staining *in vivo* and *in vitro* according to the manufacturers' instructions.

For TUNEL fluorescent staining, pancreatic tissue samples were fixed in 4% paraformaldehyde, dehydrated, embedded in paraffin, and sectioned at 4 - 5 µm. Tissue sections were deparaffinized and rehydrated. All samples were permeabilized with 0.1% Triton X - 100 in 0.1% sodium citrate. The TUNEL reaction, using a commercial kit with a mixture of TdT and fluorescein - labeled dUTP, was carried out at 37 °C for 60 min in the dark, with negative controls lacking TdT. After washing, samples were counterstained with DAPI, washed again, mounted with antifade medium, and examined under a fluorescence microscope at various magnifications. The percentage of TUNEL - positive apoptotic cells was calculated by counting green - fluorescent (TUNEL - positive) and blue - fluorescent (total, stained by DAPI) cells in multiple fields, and data were analyzed with Image J pro software, presented as mean ± SEM.

For immunofluorescence staining, pancreatic tissues obtained from approved sources were fixed in 4% paraformaldehyde for 24 h, dehydrated, cleared, embedded in paraffin, and sectioned into 4 - 5 μm slices. Paraffin - embedded tissue sections underwent deparaffinization, rehydration, and antigen retrieval. All samples were permeabilized with 0.1% Triton X - 100 and blocked with a buffer containing 5% normal serum. The primary antibody was added and incubated overnight at 4 °C, with negative controls set up. After washing, the fluorescently - labeled secondary antibody was added and incubated for 1 - 2 h at room temperature. Following another washing step, nuclei were counterstained with DAPI. Samples were mounted and observed under a fluorescence microscope. Images were captured at different magnifications, and the intensity and distribution of the fluorescent signal were analyzed using Image J pro software.

For DHE, MitoTracker and Fluo-3 AM Staining, fluorescent dyes were added directly to living cells for staining. In order to prevent cell death, DAPI was not used to label the nucleus of acinic cells *in vitro*. The fluorescent dye was incubated for 30-60 min and photographed under a confocal laser microscope. Fluorescence intensity was evaluated using Image J pro software.

Flow cytometry (FCM): For Fluo-3 AM Staining (Calcium ion fluorescent probe, S1056, Beyotime, China), fluorescent dyes were added directly to living cells for staining. The fluorescent dye was incubated for 30-60 min and the cells were analyzed with a flow cytometry (ACEA Biosciences, Inc.).

Biochemical detection: Serum amylase and lipase were completed in the automatic biochemical analyzer (Servicebio, Wuhan, CN) according to the biochemical assay kit instructions (C016-1 and A054-1, Nanjing

Jiancheng Bioengineering Institute, CN).

Enzyme-linked immunosorbent assay (ELISA): LDH levels in serum and cell supernate were detected by corresponding ELISA kits (SEB864Mu and SEB864Ra) from Cloud-Clone Corp, CN. Amylase levels in cell supernate were measured using corresponding ELISA kits (SEB454Ra) from Cloud-Clone Corp, CN. The mouse IL-6 ELISA kit (SEA079Mu, Cloud-Clone Corp USCN Life Science, Wuhan, China) and tumor necrosis factor-α (TNF-α) ELISA kit (SEA133Mu, Cloud-Clone Corp USCN Life Science, Wuhan, China) were used for the detection of the levels of IL-6 and TNF-α according to the manufacturer's instructions. In brief, the testing process is as follows: (1) Preparation of standards, reagents and samples before the experiment; (2) Add samples (standard and sample) 100 μL and incubate at 37 °C for 1 h; (3) Suck and discard, add 100 μL of detection solution A, and incubate at 37 °C for 1 h; (4) Wash the board three times; (5) Add 100 μL detection solution B and incubate at 37 °C for 30 min; (6) Wash the board 5 times; (7) Add 90 μL TMB substrate and incubate at 37 °C for 10 - 20 min; (8) Add 50 μL of termination liquid and the absorbance (O.D.) was measured at 450 nm wavelength by enzyme-labeled instrument (Bio-Rad, California, USA), and the sample concentration was calculated.

Detection of antioxidant capability: The total antioxidant capacity of pancreatic tissue or AR42J cells or mouse PACs was measured by the ferric ion reducing antioxidant power (FRAP) method using a T-AOC Assay Kit (S0116, Beyotime, China) according to the manufacturer's protocol. Sample preparation: for cell samples, about 1 million cells were collected, placed in 200 μL of cold PBS solution, homogenized or ultrasound to fully break the cells and release the antioxidants in them, centrifuged at 4 ℃ for about 12000 g for 5 min, and supernatant was taken for subsequent determination. For the tissue samples, 100 μL of cold PBS solution was added to every 20 mg of tissue, homogenized or ultrasonic to fully break the tissue and release the antioxidants in it,

centrifuged at 4 ℃ for about 12000g for 5 min, and the supernatant was taken for subsequent determination. Determination of total antioxidant capacity: Add 180 μL of FRAP working fluid to each test hole of the 96-well plate. 5 μL of PBS were added into the blank control hole. Add 5 μL of $FeSO_4$ standard solution of various concentrations into the standard curve detection hole; 5 μL of various samples or 0.15 - 1.5 mM of Trolox were added into the sample test hole as a positive control. A593 was determined after incubation at 37 ℃ for 3-5 min. The total antioxidant capacity of the sample was calculated according to the standard curve.

Western blot analysis: Pancreatic tissues were lysed in cold RIPA (P0013B, Beyotime, CN). The protein concentration was evaluated with the BCA Protein Assay Kit (P0009, Beyotime, Beijing, China). After gel electrophoresis, the protein was transferred to PVDF membrane (FFP70, Beyotime, Beijing, China) and incubation in blocking solution (3% BSA or 5% skimmed milk) at room temperature. Then the membranes were incubated overnight at 4 °C with the primary antibodies. Primary antibodies were diluted in Primary Antibody Dilution Buffer for Western Blot (P0256, Beyotime, Beijing, China). Membranes were washed and then incubated with specific HRP-conjugated secondary antibodies for 1 h at room temperature. Bands were developed using Digital gel image analysis system (Bio-Rad, California, USA) and the gray values of the bands were quantitatively analyzed by Image J software. The antibodies used in this study are listed in the *Supplementary Table* below.

Supplementary Table: Antibodies

Antibody	Item No	Company and location
HSP60 Rabbit Monoclonal Antibody	AG2237	Beyotime Biotechnology, CN
β-Actin Mouse Monoclonal Antibody	AF0003	Beyotime Biotechnology, CN
RIP3 Rabbit mAb	15828	Cell Signaling Technology, Beverly, MA, USA
Anti-Cleaved Caspase-3	ab214430	Abcam, Cambridge, MA, USA
Phospho-eIF-2α (Ser51) Antibody	AF5803	Beyotime Biotechnology, CN
Anti-PGC-1 alpha	ab191838	Abcam, Cambridge, MA, USA
Bax Rabbit mAb	14796	Cell Signaling Technology, Beverly, MA, USA
eIF2α (D7D3) XP® Rabbit mAb	5324	Cell Signaling Technology, Beverly, MA, USA
IRE1α (14C10) Rabbit mAb	3294	Cell Signaling Technology, Beverly, MA, USA
CHOP Mouse mAb	2895	Cell Signaling Technology, Beverly, MA, USA
Phospho-IRE-1α (Ser724) Antibody	AF5842	Beyotime Biotechnology, CN
BiP Antibody	3183	Cell Signaling Technology, Beverly, MA, USA
GRP78 Rabbit Polyclonal Antibody	AF0171	Beyotime Biotechnology, CN
PDI (C81H6) Rabbit mAb	3501	Cell Signaling Technology, Beverly, MA, USA
Anti-TRPM4 Antibody	ab106200	Abcam, Cambridge, MA, USA
PINK1 Rabbit Polyclonal Antibody	Af7755	Beyotime Biotechnology, CN
TRPM4 Polyclonal Antibody	PA5-116483	Thermo Fisher Scientific, ShangHai, CN
Goat anti-Mouse IgG antibody	31430	PIONEER Biotechnology, CN
Goat anti-Rabbit IgG antibody	31460	PIONEER Biotechnology, CN
Anti-LY6G antibody	ab238132	Abcam, Cambridge, MA, USA
MFN2 Rabbit Polyclonal Antibody	AF7473	Beyotime Biotechnology, CN

Figure S1. Low dose of 9-phenanthrol had little protective effect on experimental AP. (**A**) Representative photos of H&E staining of the pancreas (200X); (**B**) Pancreatic injury scores; (**C**) Percentages of necrotic areas. n = 6, error bars indicate the SEM; n.s: non-significant; AP, acute pancreatitis; LPS, lipopolysaccharide.

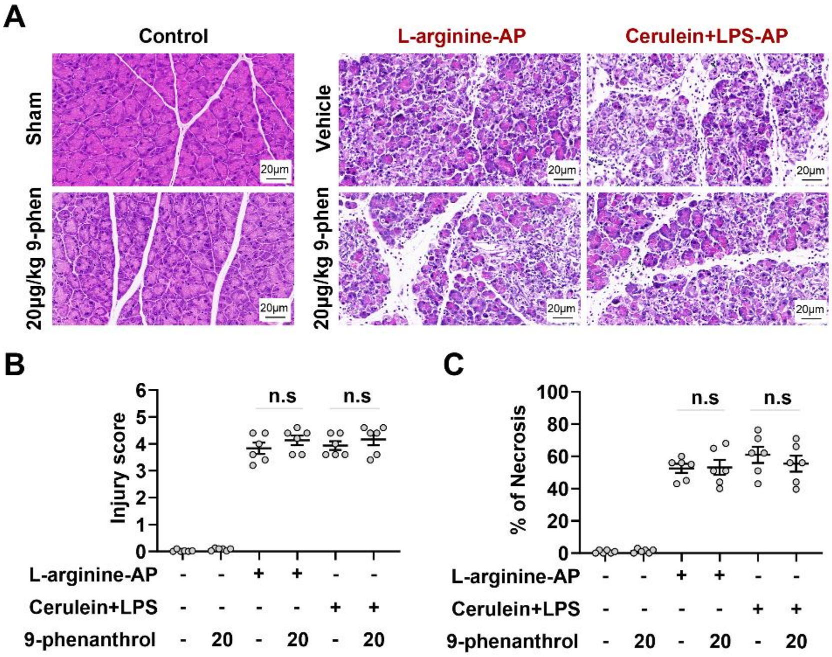

Figure S2. 9-phenanthrol alleviates inflammation in experimental AP. (**A-B**) Representative images of LY6G staining and quantitative of LY6G staining; (**C**) Serum TNF-α level; (**D**) Serum IL-6 level. n = 6, error bars indicate the SEM; ＊ P < 0.05 vs Sham or vs Control; # P < 0.05 vs Vehicle. LPS, lipopolysaccharide; TNF-α: tumor necrosis factor-α; IL-6: interleukin 6; AP, acute pancreatitis.

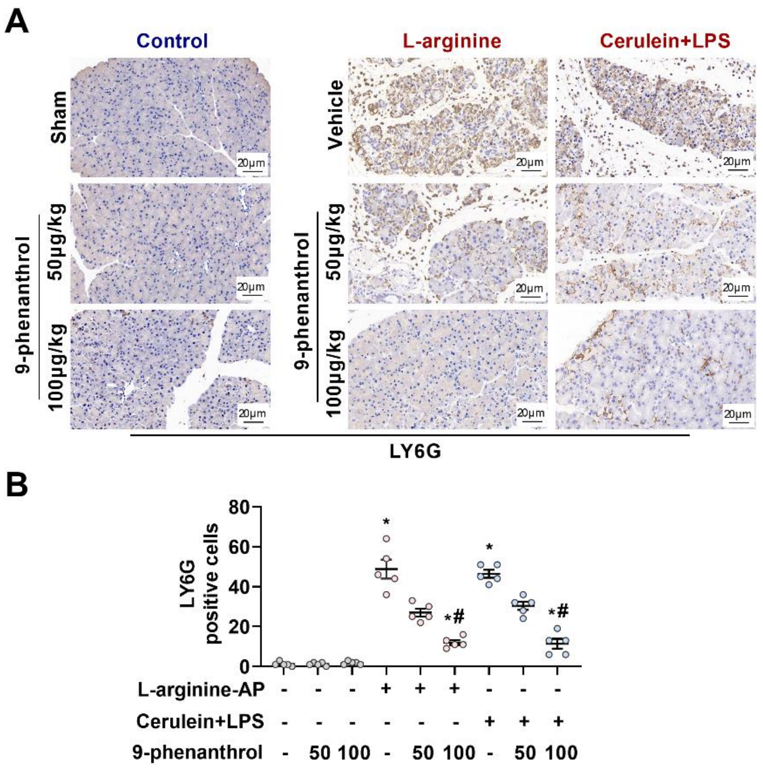

Figure S3. *Trpm4*-knockout alleviates inflammation in experimental AP. Representative photos of LY6G staining and quantitative of LY6G staining. n = 6, error bars indicate the SEM; ∗ P < 0.05 vs Sham or vs Control; # P < 0.05 vs Vehicle. LPS, lipopolysaccharide; AP, acute pancreatitis.

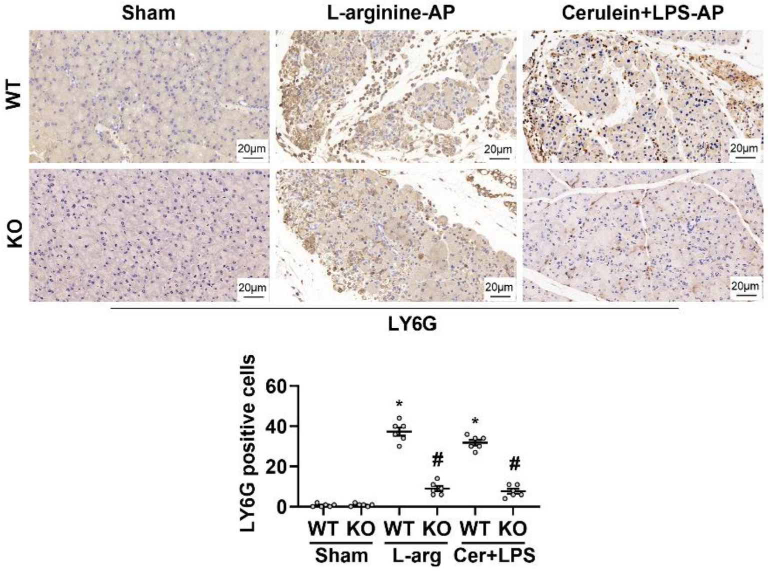

Figure S4. *Trpm4*-plasmid overexpressed TRPM4 levels in AR42J cells. Western blot analysis of the TRPM4 expression level in AR42J cells. n = 6; error bars indicate the SEM; ∗ $P < 0.05$ vs Pl-Vector. TRPM4, Transient receptor potential cation channel melastatin 4.

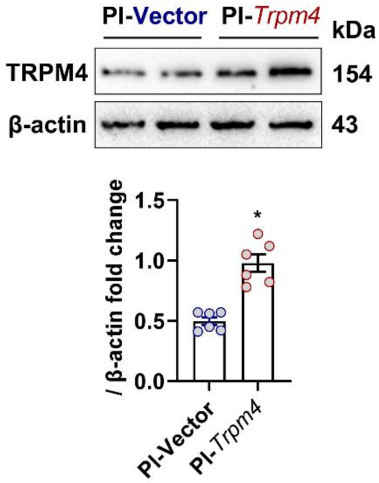

Figure S5. Inhibition of TRPM4 antagonizes mitochondrial dysfunction and cell death in acinar cells induced by Ca^{2+} overload. (**A**) Flow cytometry analysis of Fluo-3 in mouse PACs; (**B**) Supernatant LDH levels; (**C**) Western blot analysis of the TRPM4 expression level in mouse PACs; (**D**) Supernatant LDH levels; (**E**) ATP levels in mouse PACs; (**F**) FRAP levels in mouse PACs; (**G**) Flow cytometry analysis of Fluo-3 in mouse PACs. n = 3-6, error bars indicate the SEM; ∗ $P < 0.05$ vs Sham or vs Control; # $P < 0.05$ vs Vehicle. PACs, pancreatic acinar cells; LDH, lactate dehydrogenase; ATP, adenosine triphosphate; FRAP: Ferric Reducing Antioxidant Power.

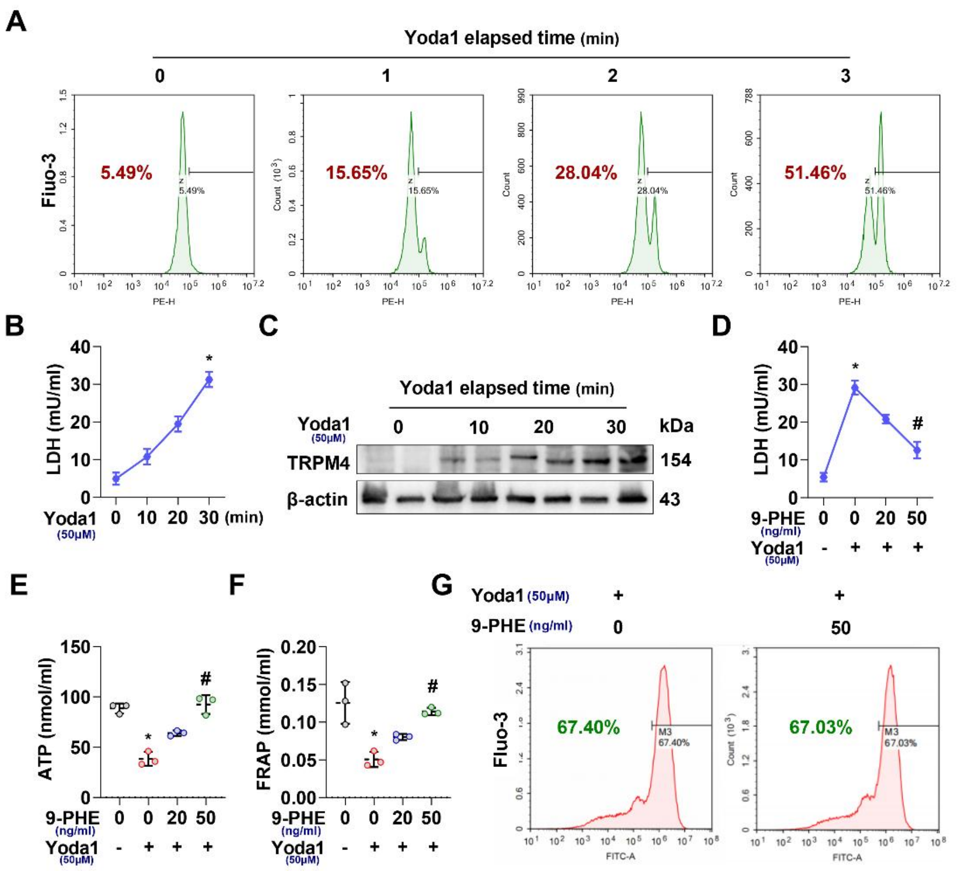

Figure S6. NMDA receptor agonists exacerbate Cerulein-induced acinar cell damage. Supernatant amylase levels. n = 6, error bars indicate the SEM. LPS, lipopolysaccharide; NMDA: N-methyl-d-aspartate.

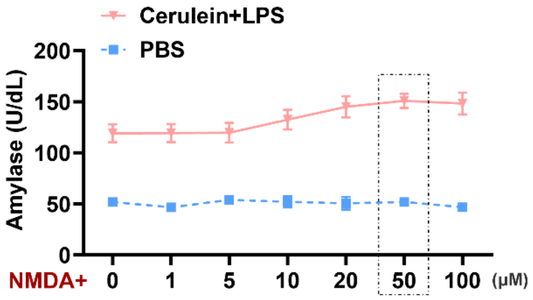

Figure S7. Inhibition of NMDAR had little effect on the expression of TRPM4 in pancreatic tissue of AP mice. (**A-B**) Western blot analysis of TRPM4 expression level in AR42J. TRPM4, Transient receptor potential cation channel melastatin 4; AP, acute pancreatitis; LPS, lipopolysaccharide.

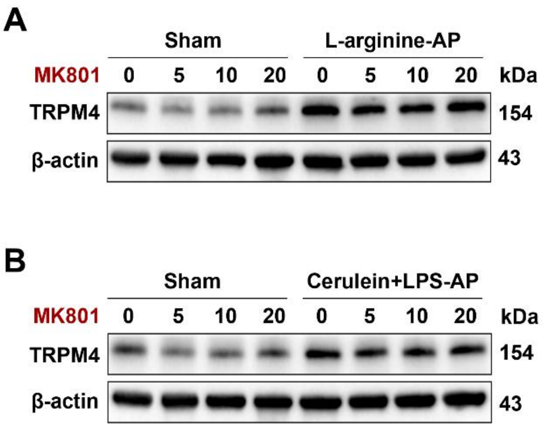

Raw western blot images

FIG-1

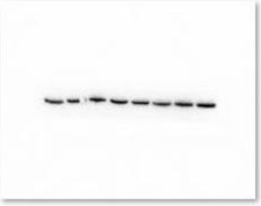
FIG-1A-ACTIN

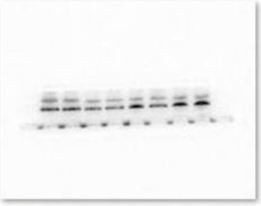
FIG-1A-TRPM4

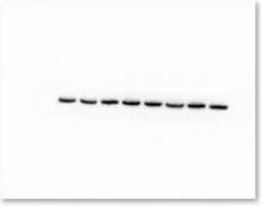
FIG-1B-ACTIN

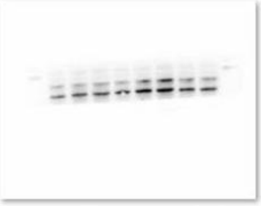
FIG-1B-TRPM4

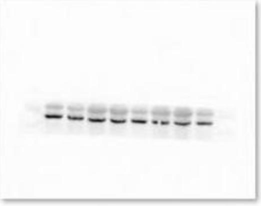
FIG-1C-ACTIN

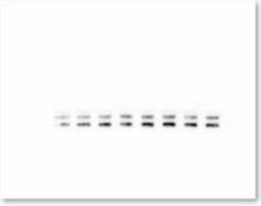
FIG-1C-TRPM4

FIG-2

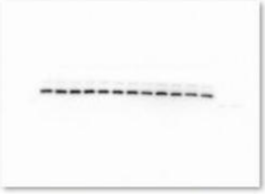
2H-ACTIN

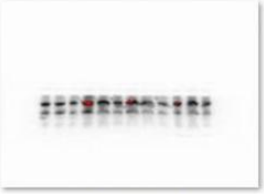
2H-RIP3

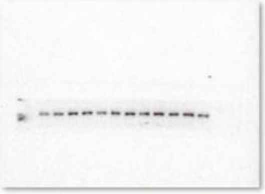
2L-ACTIN

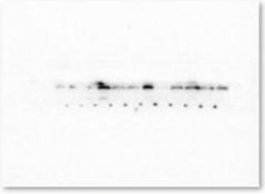
2L-BAX

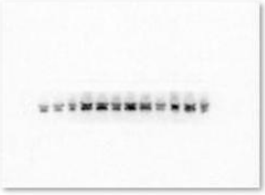
2L-CASPASE-3

FIG-3

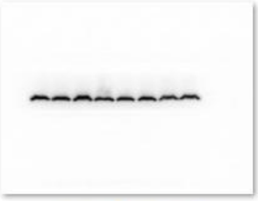
3C-ACTIN

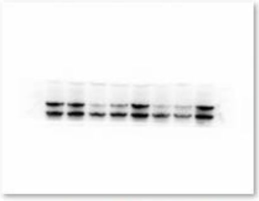
3C-MFN2

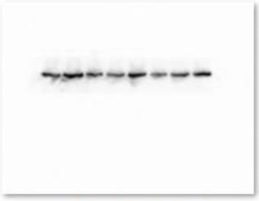
3C-PGC1

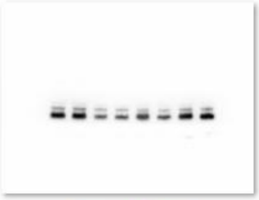
3C-PINK1

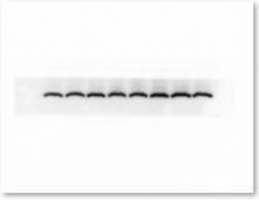
3I-ACTIN

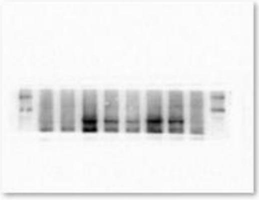
3I-GRP78

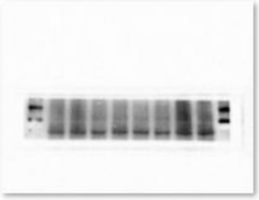
3I-IRE1

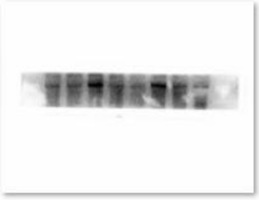
3I-p-IRE1

FIG-4

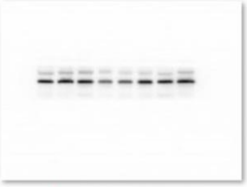
4A-ACTIN

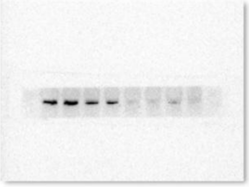
4A-TRPM4

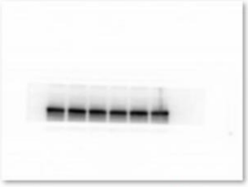
4J-ACTIN

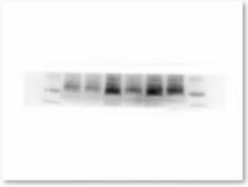
4J-RIP3

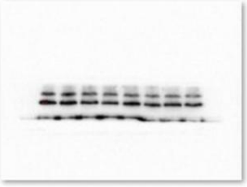
4O-ACTIN

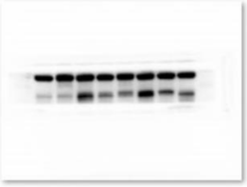
4O-GRP78

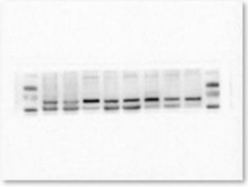
4O-PDI

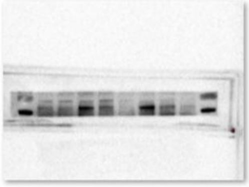
4O-P-IRE1

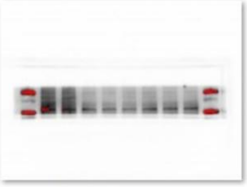
4O-T-IRE1

FIG-5

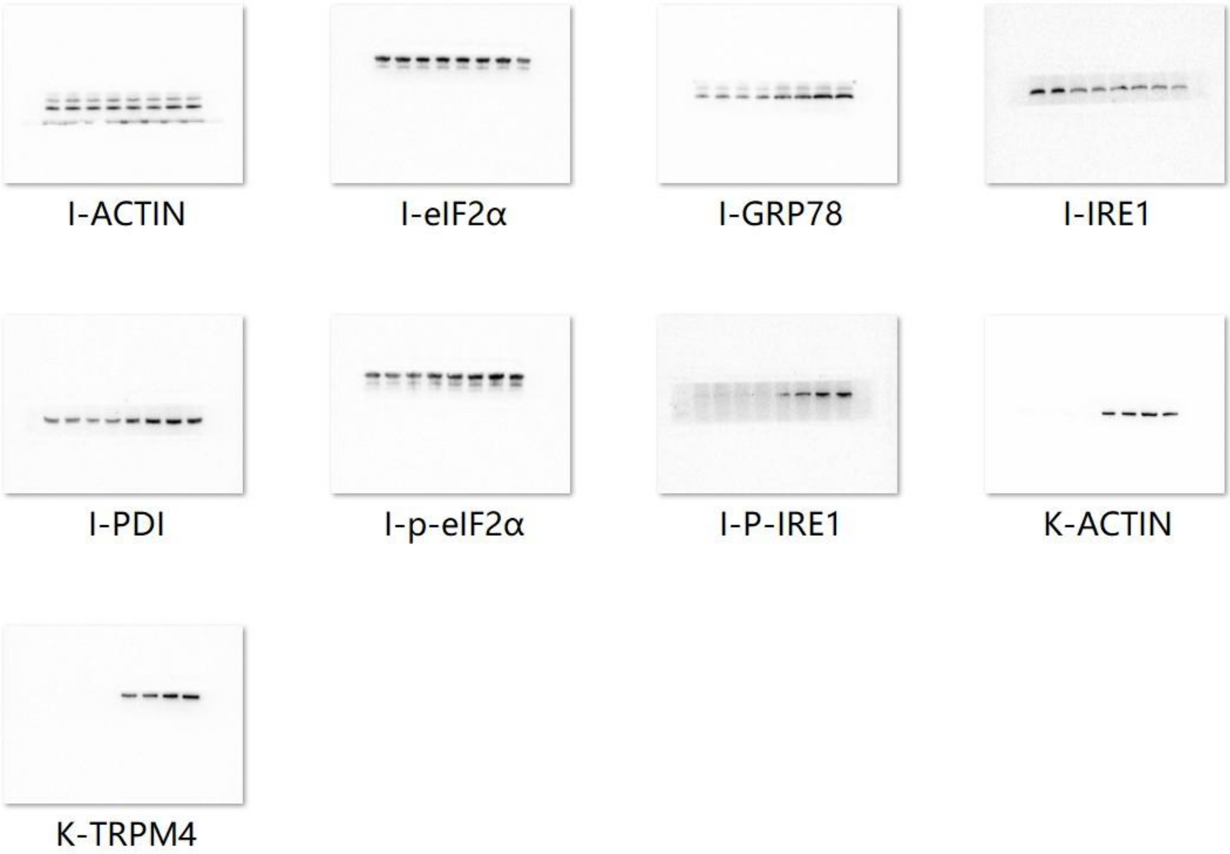
I-ACTIN
I-eIF2α
I-GRP78
I-IRE1
I-PDI
I-p-eIF2α
I-P-IRE1
K-ACTIN
K-TRPM4

FIG-6

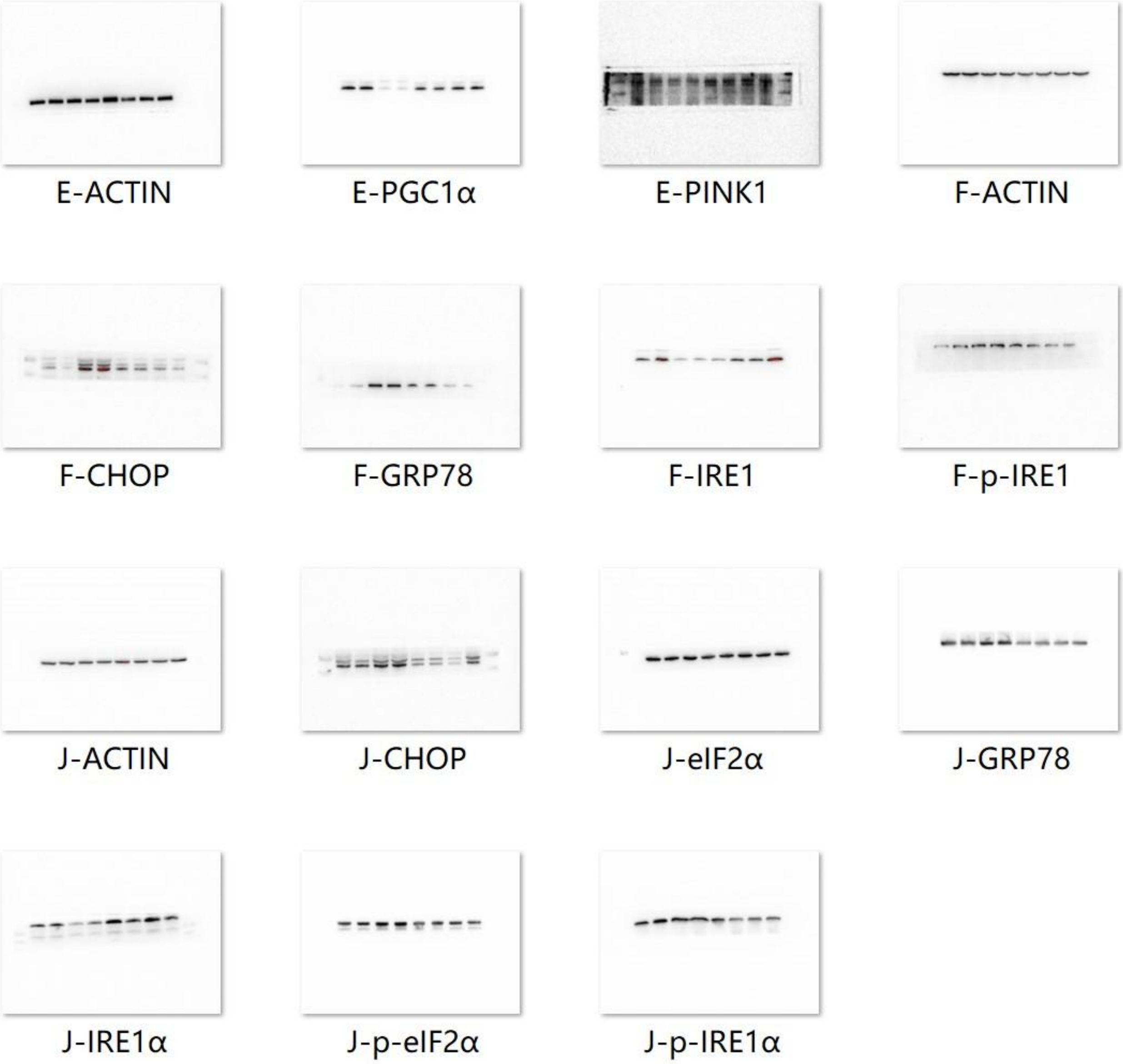
E-ACTIN
E-PGC1α
E-PINK1
F-ACTIN
F-CHOP
F-GRP78
F-IRE1
F-p-IRE1
J-ACTIN
J-CHOP
J-eIF2α
J-GRP78
J-IRE1α
J-p-eIF2α
J-p-IRE1α

FIG-7

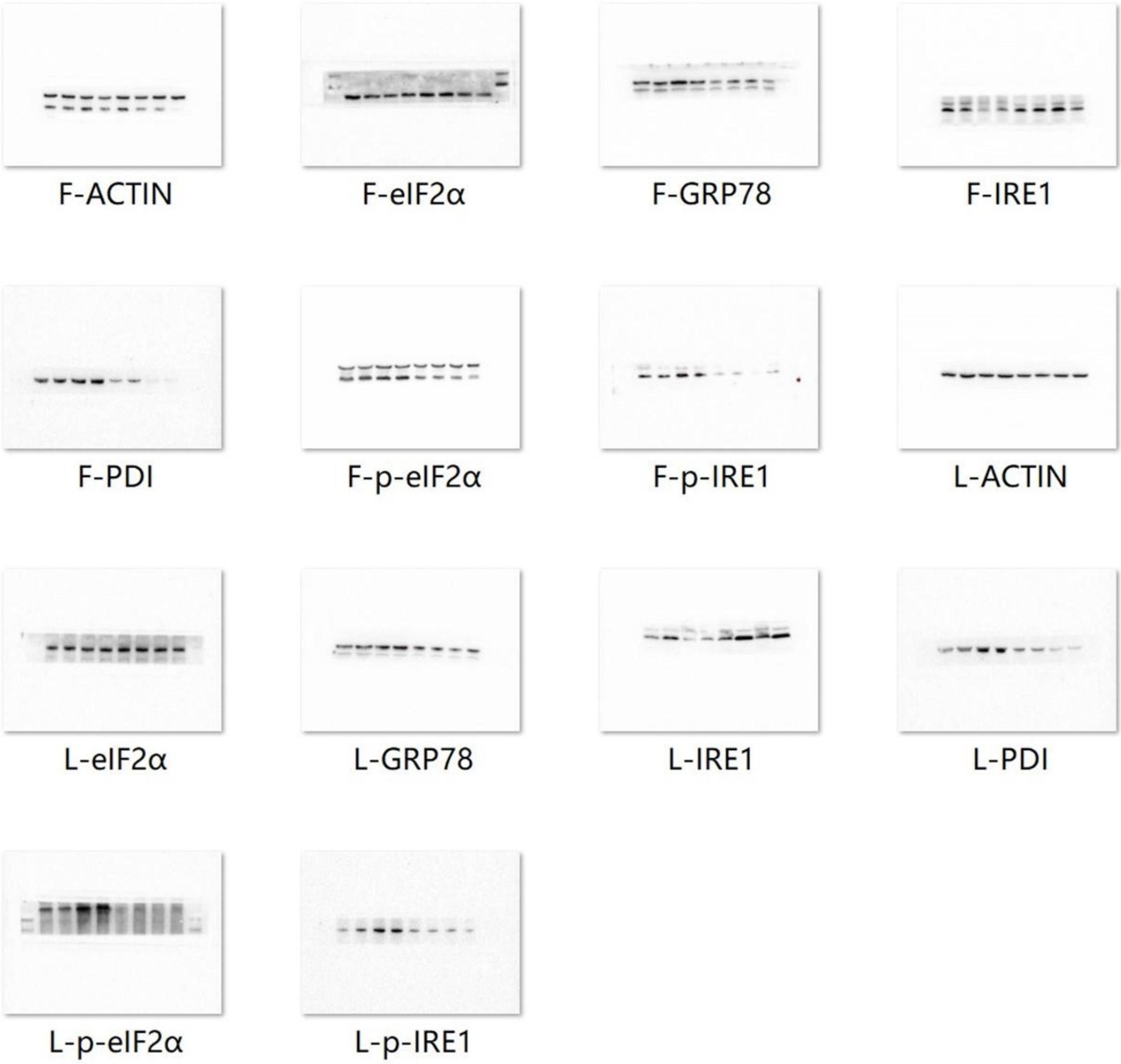
F-ACTIN
F-eIF2α
F-GRP78
F-IRE1
F-PDI
F-p-eIF2α
F-p-IRE1
L-ACTIN
L-eIF2α
L-GRP78
L-IRE1
L-PDI
L-p-eIF2α
L-p-IRE1

FIG-8

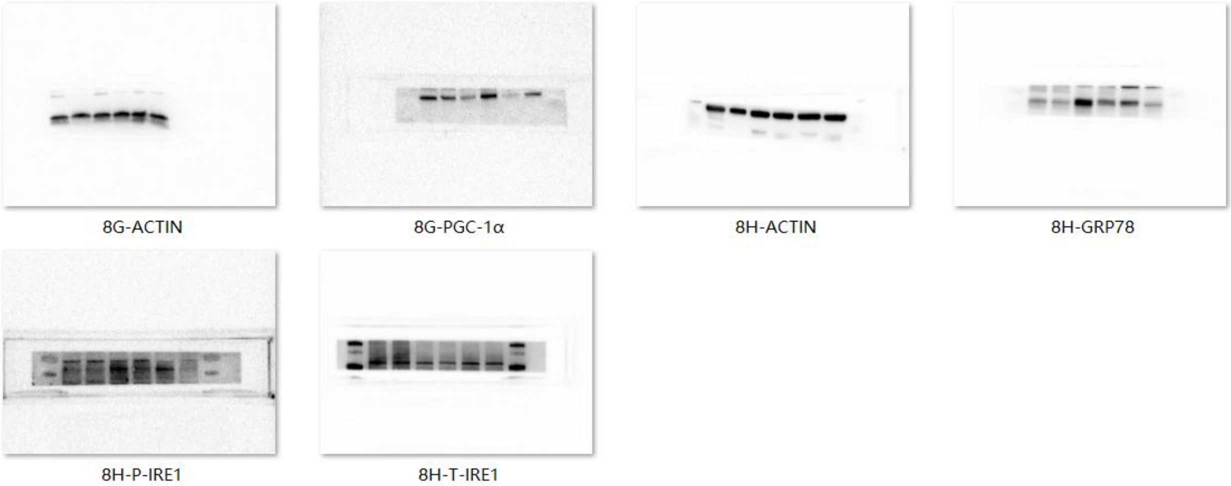

FIG-S4

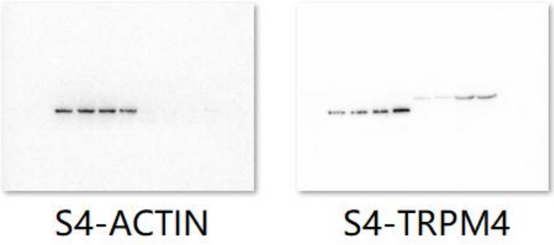

FIG-S5

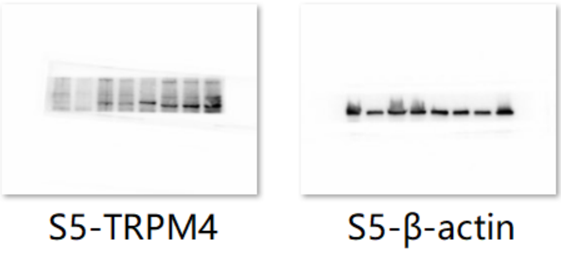

FIG-S7

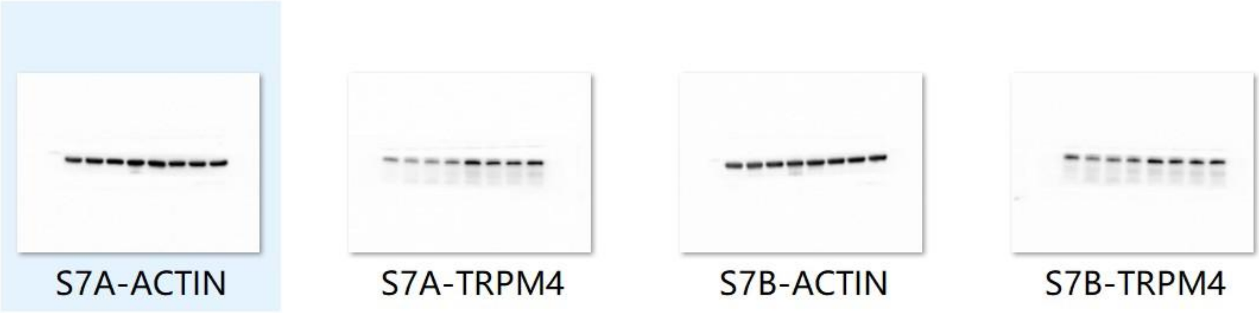

Report of *trpm4* gene knockout mice

1. Product Information

Name	C57BL/6NCya-*Trpm4*^{em1}*/Cya*
Serial Number	KOCMP-68667-Trpm4-B6N-VA
Gene	Trpm4
NCBI ID	68667
Strain	C57BL/6NCya
Type	conventional knockout

2. Targeting Strategy

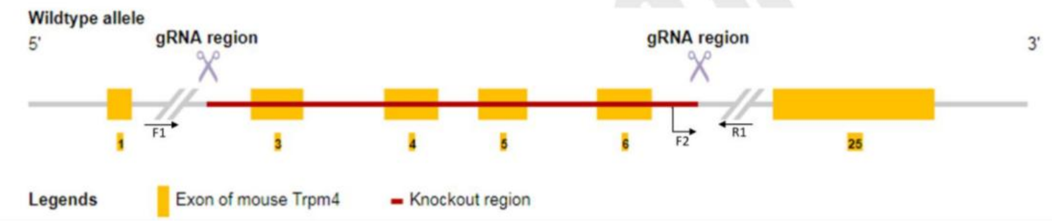

3. Genotyping strategy

<u>Primers1:</u> (Annealing Temperature 60.0 ºC)
F1: 5'-AGTCCCTGCTCATTACTCTGGG-3'
R1: 5'-GAGAAGGTAAAGAAGCCTGTGC-3'
Product size: 838 bp

<u>Primers2:</u> (Annealing Temperature 60.0 ºC)
F2: 5'-TGGGAACTTGAGGATGAAGTCAG-3'
R1: 5'-GAGAAGGTAAAGAAGCCTGTGC-3'
Product size: 643 bp

Homozygotes: one band with 838 bp
Heterozygotes: two bands with 838 bp and 643 bp
Wildtype allele: one band with 643 bp

4. Expected Results

Genotyping	Primers1	Primers2
Trpm4(-/-): Homozygotes	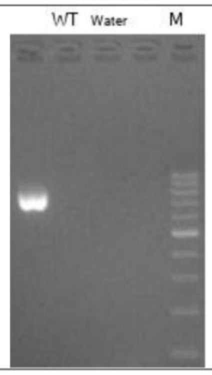	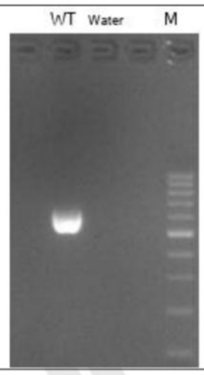

Note:

1) PCR was carried out in 25 μL volume for 35 cycles under standard conditions, with all two primers listed above added to each reaction.

2) DNA marker: Thermo Scientific GeneRuler 100 bp DNA Ladder #SM0242

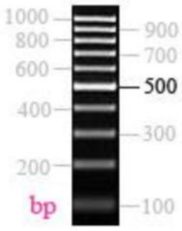

3) Controls used in PCR genotyping are:

- Water control: No DNA template added.
- Wildtype control: Mouse genomic DNA.

5. PCR reaction

5.1 DNA Extraction

➢ Method One:

We recommend that using TaKaRa MiniBEST Universal Genomic DNA Extraction kit (Ver.5.0_Code No. 9765) to gain high purity of genomic DNA.

a. Add 180 μL of Buffer GL, 20 μL of Proteinase K and 10 μL of RNase A per tail piece (2-5 mm) in a microcentrifuge tube. Be careful not to cut too much tail.

b. Incubate the tube at 56℃ overnight.

c. Spin in microcentrifuge at 12,000 rpm for 2 minutes to remove impurities.

d. Add 200 μL Buffer GB and 200 μL absolute ethyl alcohol with sufficient mixing.

e. Place the spin Column in a collection tube. Apply the sample to the spin and centrifuge at 12,000 rpm for 2 min. Discard flow-through.

f. Add 500 μL Buffer WA to the spin column and centrifuge at 12,000 rpm for 1 min. Discard flow-through.

g. Add 700 μL Buffer WB to the spin column and centrifuge at 12,000 rpm for 1 min. Discard flow-through. (Note: Make sure the Buffer WB has been premixed with 100% ethanol. When adding Buffer WB, add to the tube wall to wash off the residual salt.)

h. Repeat step g.

i. Place the spin Column in a collection tube and centrifuge at 12,000 rpm for 2 min.

j. Place the spin Column in a new 1.5ml tube. Add 50~200 μL sterilized water or elution buffer to the center of the column membrane and let the column stand 5min. (Note: Heating sterilized water or elution buffer up to 65℃ can increase the yield of elution.)

k. To elute DNA, centrifuge the column at 12,000 rpm for 2 min. To increase the yield of DNA, add the flow-through and/or 50~200 μL sterilized water or elution buffer to the center of the spin column membrane and let the column stand 5 min. Centrifuge at 12,000 rpm for 2 min.

l. Quantify to genomic DNA. Eluted genomic DNA can be quantified by electrophoresis or absorbance.

➢ Method Two:

A low-cost and sample method to gain rough genomic DNA.

a. Add 100 μL of tail digestion buffer per tail piece (2-5 mm) in a microcentrifuge tube. Be careful not to cut too much tail.

b. Incubate the tube at 56℃ overnight.

c. Incubate the tube at 98℃ for 13 minutes to denature the Proteinase K.

d. Spin in microcentrifuge at top speed for 15 minutes. Use an aliquot of supernatant straight from the tube (1 μL in a 12.5 μL reaction) for PCR.

Final concentration of tail digestion buffer:

➢ 50 mM KCl

➢ 10 mM Tris-HCl (pH 9.0)

- 0.1 % Triton X-100
- 0.4 mg/mL Proteinase K

5.2 PCR Mixture (primer concentration: 10μM):

Component	x1
ddH_2O	9.0 μl
Product primer F	1.0 μl
Product primer R	1.0 μl
Premix Taq	12.5 μl
DNA	1.5 μl
Total	25 μl

5.3 PCR Reaction Conditions:

Step	Temp.	Time	Cycles
Initial denaturation	94 °C	3 min	
Denaturation	94 °C	30 s	
Annealing	60 °C	35 s	35 x
Extension	72 °C	35 s	
Additional extension	72 °C	5 min	

5.4 Relevant Reagents:

**Trizma Hydrochloride Solution**	Sigma, Cat. No. T2663
**Proteinase K**	Merck, Cat. No. MK539480
**Triton X-100**	Sigma, T8787-50 mL
**Premix Taq Polymerase**	Vazyme, P222
**Agarose**	BIOWEST AGAROSE, REGULAR
**DNA Marker**	Thermo Scientific GeneRuler 100 bp DNA Ladder #SM0242
**0.5×TBE**	Tris Bio Basic Inc, TBO194-500g
	EDTA Shanghai Sangon, 0105-500g
	Boric Acid, Shanghai Sangon, 0588-500g

Cyagen help you discover life
